# Integrative multi-omics identification and functional validation of potential targets linking metabolism–immune–colorectal cancer causal pathway

**DOI:** 10.3389/fimmu.2025.1649788

**Published:** 2025-09-08

**Authors:** Zequn Zheng, Xiaoling Xu

**Affiliations:** ^1^ The Affiliated Lihuili Hospital of Ningbo University, Health Science Center, Ningbo University, Ningbo, Zhejiang, China; ^2^ Department of General Surgery, First Affiliated Hospital of Shantou University Medical College, Shantou, Guangdong, China

**Keywords:** colorectal cancer (CRC), multiomics, omega-3 polyunsaturated fatty acids, CD4^+^ T cell, SLC6A19

## Abstract

**Introduction:**

Colorectal cancer (CRC) remains a major global health burden, highlighting the need for novel molecular targets for therapy and prognosis. This study integrates multi-omics data with functional assays to explore metabolite-mediated mechanisms in CRC risk.

**Methods:**

We performed genetic causal inference and colocalization analyses using genome-wide association data to assess causality between 233 metabolites and CRC. A total of 731 immune traits were investigated as potential mediators. Metabolite-associated CpG sites were identified via epigenome-wide association studies (EWAS), and their methylation QTLs (mQTLs) were linked to target genes through interaction eQTL analysis via FUMAGWAS. Expression, prognosis, immune infiltration, and regulatory associations of target genes were analyzed using TCGA datasets. Functional assays were conducted in NCM460 and CRC cell lines (HCT116, SW480, CACO2). CRC xenograft mice were used to monitor tumor growth *in vivo*.

**Results:**

A higher omega-3 fatty acid ratio (FAw3byFA, OR = 1.22, P = 2.51×10^-7^) was associated with increased CRC risk, with partial mediation (10%) via Effector Memory CD4^+^ T cells. Colocalization (PP.H4 ≈ 0.97) suggested shared genetic loci. Genetically predicted omega-3-associated CpG sites, cg05181941, cg06817802, and cg22456785, were linked to CRC risk. These sites-derived 428 mQTLs interact with eQTL genes, highlighting SLC6A19 as a potential target, expressed in CD4^+^ T cells , colon tissue and CRC epithelial cells. SLC6A19 was downregulated in TCGA-COAD, -READ, and -COADREAD and confirmed by immunoblotting, correlating with poor survival and CD4^+^ T cell infiltration. CCK-8, wound healing, and Transwell assays showed that SLC6A19 overexpression suppressed CRC cell proliferation, migration, and invasion. *In vivo*, SLC6A19 overexpression significantly reduced CRC xenograft tumor growth.

**Conclusions:**

Omega-3-related methylation-intersecting SLC6A19 potentially mediates omega-3-CD4^+^ T cells-driven CRC risk, suggesting a candidate inhibitory target.

## Graphical Abstract

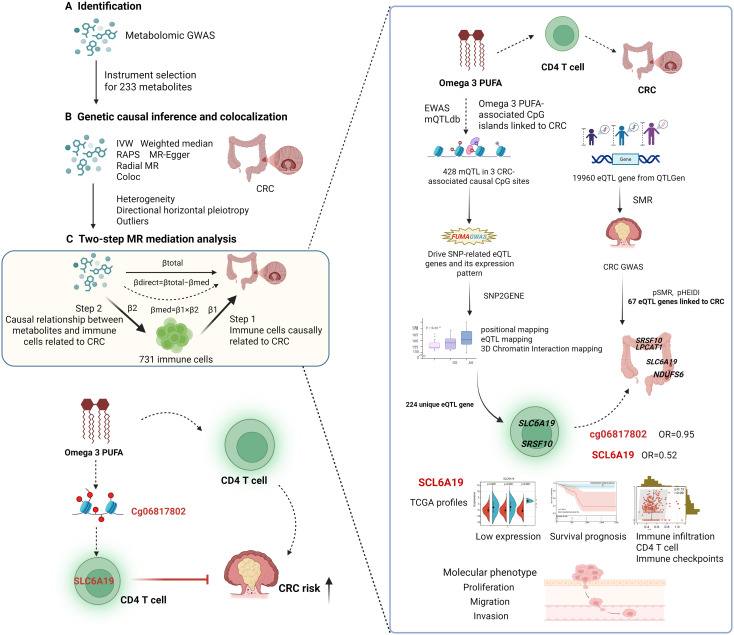

## Introduction

Consistently ranked the second biggest killer of cancer globally and third most frequent neoplasm, colorectal cancer (CRC) is a principal cause of world cancer morbidity and mortality (1, 2). In 2022, there were an estimated 1.9 million new CRC cases and 904,000 deaths worldwide and early-onset CRC (EOCRC) is rising across most regions: incidence is rising across 27 out of 50 countries examined, most often faster than for older adults (3, 4). Despite improvements in diagnostic screening and treatment methods, the overall illness burden remains high, especially in low- and middle-income nations, mirroring changing epidemiology not yet well addressed by current screening and preventive measures (5, 6).

A growing body of work suggests that host metabolism and immunity co-shape CRC risk and progression through effects on the tumor microenvironment (7–9). The relationship between fatty acid metabolism and CRC may be more intricate than previously assumed, involving context-dependent effects mediated by genetic, epigenetic, and immune factors (10–13). Among metabolic inputs, polyunsaturated fatty acids (PUFAs), especially omega-3 (n-3) species, have attracted intense interest for their capacity to remodel membrane composition, signaling, and T-cell responses, as well as to generate specialized pro-resolving mediators (e.g., resolvins, protectins) that can recalibrate inflammation (13–18). However, human data linking n-3 PUFAs to CRC are inconsistent, with observational studies susceptible to confounding and reverse causation. Mendelian randomization (MR) study results have presented inverse associations between CRC risk and shorter-chain n-3 or n-6 fatty acids, such as α-linolenic acid (ALA, n-3) and linoleic acid (LA, n-6) (19). Observational studies present findings where higher dietary intakes of EPA, DHA, and DPA are inversely associated with CRC risk, and where a high dietary n-6/n-3 PUFA and a high dietary intake of trans fat are correlated with elevated risk (20). Such contradictions imply species- and situation-dependent actions deserving detailed mechanistic analysis. Epigenetic regulation offers a viable connection between exposure to fatty acids and stable transcription programs of relevance to carcinogenesis. Changes in DNA methylation are evident early during CRC and form the basis of commonly utilized biomarkers like SEPT9, demonstrating their biological and clinical significance (21). Moreover, n-3 PUFAs can be modulated by DNA methylation in immune and peripheral blood cells, implying that lipid exposures may reprogram immune function via epigenetic mechanisms (22). Yet the direction, tissue specificity, and causal relevance of n-3-related methylation changes for CRC risk remain unresolved (23, 24). On the immune side, n-3-derived mediators and membrane remodeling can influence CD4^+^ T-cell differentiation, effector function, and exhaustion, but how n-3 composition maps onto specific T-cell compartments that shape CRC susceptibility is unclear (15–17, 25). Defining which immune phenotypes lie on the causal pathway from circulating fatty acids to CRC and through which epigenetic and transcriptional nodes remain a central gap.

To address these knowledge gaps, we conducted a multi-omics investigation integrating genome-wide association data, epigenome-wide DNA methylation profiles, transcriptomic signatures, metabolite quantifications, immunophenotyping, quantitative trait loci (QTL) mapping, and tumor datasets from The Cancer Genome Atlas (TCGA). Employing causality-based MR, colocalization analysis, mediation models, transcriptome-wide association studies, survival analysis, immune infiltration profiling, and molecular functional assays (26–29). Complemented by targeted functional assays, we aim to pinpoint tractable nodes in the metabolism-immunity axis of CRC.

## Methods

### Study design and analytical strategy

This study employed a comprehensive multi-omics approach to investigate the causal pathways linking circulating metabolites with CRC risk through immune cell mediation. The analysis began with metabolome-wide association studies using large-scale GWAS data to identify 233 metabolite candidates, followed by rigorous MR analyses with sensitivity testing to establish causal relationships with CRC susceptibility. Colocalization analysis was further conducted to verify shared causal variants between metabolites and CRC risk. To elucidate potential immune mechanisms, we implemented a novel two-step analytical framework (26, 30): first identifying CRC-associated immune cell traits from 731 immunophenotypes, then examining whether causal metabolites influence these specific immune characteristics. This approach revealed critical immunological mediators in the metabolite-CRC axis. To further characterize epigenetic mechanisms, we retrieved epigenome-wide association study (EWAS) data for the identified metabolites and mapped their associated CpG sites. Methylation QTLs (mQTLs) corresponding to these CpG sites were used as instruments in MR analyses to identify metabolite-driven CpG methylation sites associated with CRC risk. To investigate potential transcriptomic links between metabolites and CRC, we obtained expression quantitative trait loci (eQTL) data for 19,960 genes from the eQTLGen consortium. We applied Summary-data-based MR (SMR) combined with the HEIDI test to prioritize candidate genes associated with CRC. These genes were intersected with eQTL genes driven by metabolite-related mQTLs, identified using the FUMA GWAS platform. Genes present in both datasets were considered as potential overlapping targets mediating CRC risk. Finally, to functionally validate the candidate target genes, we analyzed their expression patterns, prognostic relevance, and immune-related features using the TCGA CRC dataset. *In vitro* and *in vivo* experiments were conducted to confirm their roles in CRC-relevant phenotypes. This systematic strategy bridges metabolic dysregulation, immune modulation, and CRC pathogenesis through vertically integrated omics layers, providing mechanistic insights into cancer metabolic immunology (**Graphical Abstract**).

### Sources of genetic data for studied phenotypes

Metabolite instruments came from Karjalainen et al.’s NMR-based GWAS of 136,016 Europeans (171 lipoproteins, 23 lipids, 19 fatty acids, 20 other metabolites; GCST90301941 - 90302173) (29). Files were harmonized in R for MR. Immune-trait GWAS (731 phenotypes, n = 3,757) were drawn from a Sardinian cohort (IEU IDs ebi-a-GCST90001391–90002121) (31). CRC summary statistics (8,801 cases; 345,118 controls) were obtained from FinnGen Release 11 (“finngen_R11_C3_COLORECTAL_EXALLC”) with non-cancer controls. Omega-3 PUFA EWAS data (670 samples; trait EFO_0005110) and ARIES mQTLs provided methylation instruments (± 1 Mb). eQTLGen (n = 31,684) supplied cis-eQTLs. This dataset provides high-resolution cis-eQTL mappings derived from blood RNA, enabling reliable identification of genetic variants regulating gene expression (32). TCGA (COAD, READ, COADREAD) furnished tumor expression, immune-gene profiles, and survival data; samples with <30-day follow-up were excluded (33). All datasets comply with original ethical approvals; no additional consent was required for these secondary analyses.

### Genetic instrumentation of exposures

We assessed causal relationships between circulating metabolites, immune cell traits, epigenetic CpG loci (exposures), and CRC (outcome) using genetic instruments as proxies. The TwoSampleMR package (v0.6.14) was used for data preprocessing and MR analysis. CpG-related mQTLs served directly as IVs. For metabolites, SNPs were filtered using a Bonferroni-corrected threshold (P < 1.8 × 10⁻^9^), derived by dividing P < 5 × 10⁻^8^ by 28 principal components explaining >95% of trait variance. This corrected for over-conservative adjustments in multi-phenotype GWAS (27, 29). Independence was ensured by retaining SNPs with r² < 0.001 within 10 Mb windows. Weak instruments (F-statistics < 10) were excluded, and traits with fewer than three instruments were removed to maintain power, due to high intercorrelation in NMR data. To reduce confounding, SNPs associated with CRC risk factors (e.g., alcohol, smoking, BMI, diabetes) were removed using LDlink (34).

Outcome associations were retrieved with extract_outcome_data(), and data harmonized using harmonise_data() to align effect alleles. Ambiguous SNPs (e.g., A/T, C/G) were handled cautiously, with palindromic variants near allele frequency 0.5 excluded (action = 2) to avoid strand ambiguity. MR estimates were then computed, with multiple sensitivity analyses conducted to assess robustness.

### Statistical analysis

Causal relationship for circulating metabolites and CRC: To assess the causal impact of circulating metabolites on CRC, we applied MR methods. For exposures with multiple IVs, we used inverse variance weighted with multiplicative random-effects (IVW-MRE), assuming IV validity and independence. When only a single SNP was available, we used the Wald ratio (30). Given IVW’s sensitivity to directional pleiotropy, we also applied MR-Egger, weighted median, and radial MR to improve reliability. Additionally, RAPS (mr.raps v0.4.1) was used to adjust for weak instruments and pleiotropic bias (35). To address multiple testing across metabolites, Bonferroni correction was used; the significance threshold was P < 0.05 divided by the number of traits in each category. Associations passing this threshold in IVW or Wald ratio analyses were considered suggestive, and if confirmed in sensitivity tests, deemed robust. Significant metabolites were further tested for colocalization with CRC (coloc v5.2.3) (36). Strong evidence of shared genetic architecture was defined as PP.H4 > 0.90.

Two-step (Mediated) MR estimation (immune cells as mediators): To examine whether immune traits mediate metabolite effects on CRC, we used a two-step MR strategy (26, 30, 37):

Step 1: Identify immune traits causally linked to CRC (effect size = β1).

Step 2: Assess if these immune traits are influenced by CRC-associated metabolites (effect size = β2).

Assuming linear relationships, the indirect (mediated) effect of the metabolite on CRC through the immune trait was computed as: βmed=β1×β2. The direct (unmediated) effect was estimated as: βdirect=βtotal−βmed (Graphic abstract). This two-stage approach allowed us to identify metabolite-immune-CRC pathways.

Epigenetic MR: CpG methylation effects on CRC were analyzed via the Wald ratio for single-SNP sites or IVW-MRE for multiple IVs, supported by weighted median and MR-PRESSO. A Bonferroni threshold of P < 0.002 (0.05/23) determined significance.

SMR and FUMAGWAS integration: We integrated eQTL and CRC GWAS data using SMR (v1.3.1) to identify candidate genes. SNPs with MAF < 0.01 were excluded, and only cis-eQTLs (P < 5 × 10⁻^8^) within ±2 Mb were considered. Significant probes passed SMR testing and Bonferroni-adjusted HEIDI tests (P_HEIDI > 0.05). In parallel, FUMAGWAS SNP2GENE was used to map Omega-3 PUFA mQTL-linked genes. Mapping employed 1000 Genomes EUR data with positional, eQTL (P < 0.05), and 3D chromatin interaction strategies. The final gene list included those identified by both SMR and FUMA.

### Sensitivity analysis

MR-based causal inference can be biased by directional pleiotropy, particularly in omics research. To evaluate robustness, we assessed instrument heterogeneity via mr_heterogeneity(), which tests for variability among instruments that could violate MR assumptions. Directional pleiotropy was tested using the MR-Egger intercept (mr_pleiotropy_test()), with P > 0.05 indicating low pleiotropic bias. To further detect and correct pleiotropic effects, we applied Radial MR (RadialMR v1.1), using both IVW and MR-Egger estimates. Radial plots helped identify and exclude outlier SNPs violating MR assumptions (38). The leave-one-out analysis was conducted to assess the significance of the driving effect of individual IVs.

### Differential expression, prognostic, diagnostic and immunoregulatory analyses

Tumor vs. normal gene expression was analyzed across cancers using unpaired Wilcoxon rank-sum and signed-rank tests. Prognostic relevance was assessed via Cox proportional hazards models (coxph from survival v3.2-7), with log-rank tests for significance. The “pROC” package was also utilized to create receiver operating characteristic (ROC) curves and to calculate area under the curve (AUC). For combined diagnostic performance, a multivariable logistic regression model was utilized to create an integrated ROC curve. With the “decision_curve” function from the “rmda” package, decision curve analysis (DCA) was conducted, including for single-gene models and for multigene models integrating two genes. The analysis was under the assumption of a case–control study design, and probability thresholds from 0 to 1 were assessed. For immune profiling, gene expression was mapped to gene symbols, and immune cell infiltration scores (e.g., B cells, CD4^+^, CD8^+^ T cells) were computed using TIMER and deconvo_CIBERSORT (IOBR package). We also analyzed expression of SLC6A19 and 60 immune checkpoint markers (24 inhibitory, 36 stimulatory) (39), along with 150 immune pathway genes (41 chemokines, 18 receptors, 21 MHCs, 24 inhibitors, 46 stimulators). Pearson correlations between SLC6A19 and these immune genes were computed. All workflows (data harmonization, MR, and outlier filtering) were performed in R v4.3.1 with a reproducible pipeline.

### Single cell pipeline and cell model construction and immunoblotting

The single-cell landscape of *SLC6A19* in CRC was profiled with the GEO dataset GSE166555. All preprocessing and downstream analyses were carried out in Seurat v5.1.0. After SCTransform normalization, highly variable genes were identified and subjected to linear dimensionality reduction; the first 20 principal components were then embedded in two dimensions with UMAP. Cluster-specific differentially expressed genes were obtained with FindAllMarkers and manually cross-checked against the CellMarker 2.0 reference sets. Gene-level expression values were extracted with FetchData function. Human intestinal epithelial cells NCM460 and CRC cell lines HCT116, SW480, and CACO2 were obtained from Procell (China). NCM460, HCT116, and SW480 were cultured in DMEM with 10% FBS, while CACO2 cells were maintained in DMEM/F-12 with 15% FBS. All cells were incubated at 37°C in 5% CO_2_ and authenticated by STR profiling within the last three years. For SLC6A19 knockdown, three siRNAs and a non-targeting control (si-NC) were used (GenePharma, China). Cells (5 × 10^5^/well) were seeded in 6-well plates and transfected at 70 – 80% confluence with 50 nM siRNA and 5 μL siRNA-mate plus in 250 μL Opti-MEM. After a 15-min incubation, the mix was added to cells; media was refreshed at 6 h, and incubation continued for 48 h. Knockdown efficiency was verified by RT-qPCR. For overexpression, 2 μg of pcDNA3.1-SLC6A19 plasmid was transfected with 5 μL GP-transfect-Mate in 250 μL Opti-MEM. After 6 h, media was changed, followed by 48 h of incubation. Controls included si-NC and empty vectors. Western blotting was performed on denatured lysates using SDS-PAGE and PVDF membranes (0.45 μm, Millipore). Membranes were blocked in 5% milk/TBST, probed with anti-SLC6A19 (Proteintech, Cat# 27575 - 1-AP, 1:1000) and HRP-conjugated secondary antibody (Cat# SA00001 - 2, 1:3000). Protein bands were visualized with ECL reagents.

### CCK-8, wound healing, migration, and invasion assays, and *in vivo* xenograft model

For the Cell Counting Kit-8 (CCK - 8) assay, transfected CRC cells were seeded into 96-well plates at a density of 2,000 cells/well, with six replicates per group. At 0, 12, 24, and 48 hours, 10 μL of CCK - 8 reagent (Dojindo, Japan) was added to each well, and absorbance at 450 nm was measured after 2 hours of incubation to assess cell viability. Wound healing assays were performed by scratching monolayers of overexpressing cells at 90% confluence in 6-well plates using a 200 μL pipette tip. After PBS washing, serum-free medium was added, and wound closure was imaged at 0 and 48 hours under an inverted microscope. For Transwell migration and invasion assays, cells were serum-starved for 12 hours, trypsinized, and resuspended in serum-free medium at 4 × 10^4^ cells/mL. For migration, 300 μL of cell suspension was added to the upper chamber (8 μm pore size; Corning) with 600 μL of medium containing 20% FBS in the lower chamber. For invasion, 100 μL of Matrigel (Corning) was pre-coated on the upper chamber membrane and solidified at 37°C for 1 – 2 hours. After 24 hours, cells were fixed with 4% paraformaldehyde, stained with 0.1% crystal violet, imaged, and counted. All *in-vivo* protocols were approved by the Institutional Animal Care and Use Committee of Shantou University Medical College. Six-week-old male BALB/c nude mice were kept under specific-pathogen-free conditions. Two HCT116 derivatives, one bearing an empty control vector (HCT116-Vector) and the other engineered to over-express *SLC6A19* (HCT116-OE-SLC6A119), were injected subcutaneously at 5×10^8^ cells in 150 µL (n = 4 per group). Animals were sacrificed 1 – 4 weeks after implantation. Tumors were excised, fixed in 10% neutral-buffered formalin for SLC6A19 immunohistochemistry, and portions were snap-frozen at -80°C for further analyses. The entire experiment was repeated independently on three occasions. Statistical analyses were performed using GraphPad Prism (version 10.4). Two-group comparisons were analyzed using independent sample t-tests, while comparisons among multiple groups were conducted using one-way ANOVA. A P-value < 0.05 was considered statistically significant.

## Results

### Metabolites with causal associations to CRC risk

We retrieved genetic IVs for 233 circulating metabolites. Two traits—conjugated linoleic acid to total fatty acids (CLAbyFA) and glycerol—were excluded due to having only two valid instruments, leaving 231 metabolites for MR analysis. These included 171 lipoproteins, 23 lipids, 18 fatty acids, and 19 amino acid/other metabolites, each represented by 3 – 81 IVs (**Supplementary Table 1**). IV strength was supported by F-statistics: 36.18 – 2152.62 (lipoproteins), 36.19 – 1915.87 (lipids), 36.19 – 5088.20 (fatty acids), and 36.33 – 7610.08 (amino acid/others), all well above the threshold of 10.

**Figure 1:**
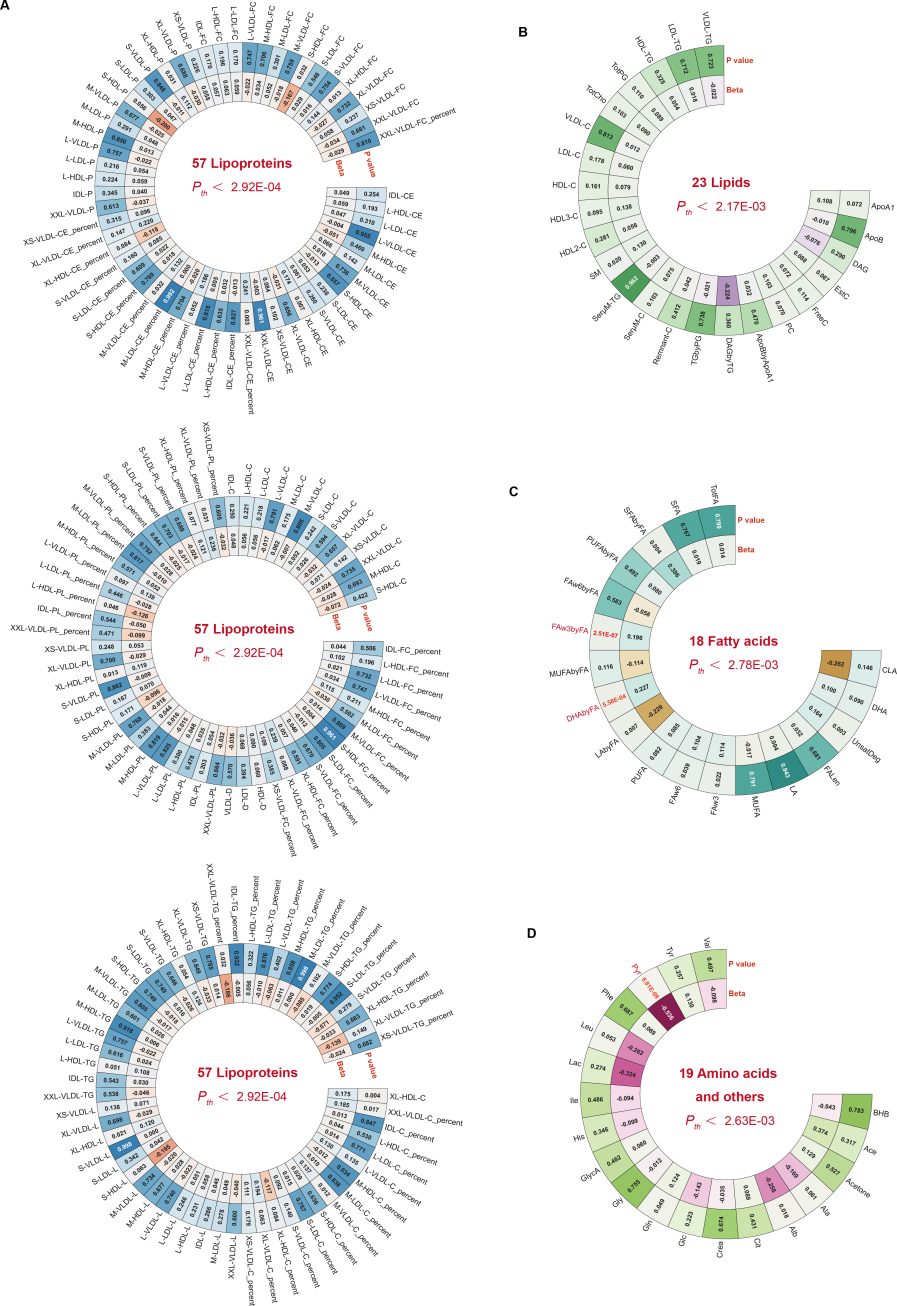
Circular heatmaps summarizing metabolite subclass associations with CRC. **(A–D)** Four panels show 171 lipoproteins, 23 lipids, 18 fatty acids, and 19 amino acids/others. Bonferroni-significant traits are shown in red.

**Figure 2:**
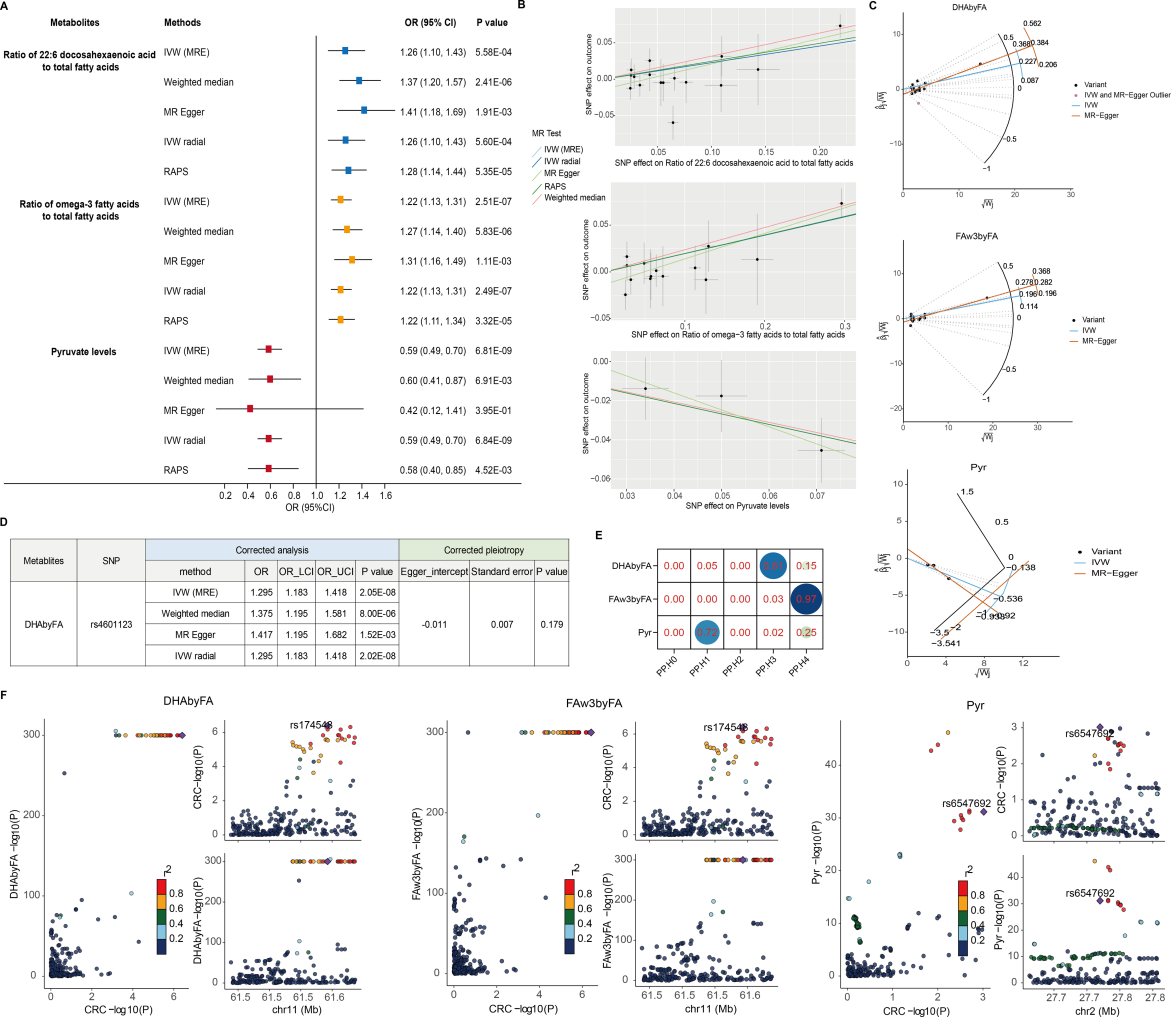
Sensitivity and colocalization analysis of metabolite–CRC links. **(A)** Forest plot of five MR methods for key metabolites. **(B)** SNP-level scatter plots for metabolite–CRC effects. **(C)** Radial MR plots detect outlier SNPs. **(D)** Corrected estimates post-outlier removal. **(E)** Heatmap of colocalization probabilities (PP.H4 > 0.90 = strong evidence). **(F)** Regional association plots via LocusCompare.

Causal effects on CRC risk were estimated using MR IVW. No lipoproteins met the Bonferroni threshold (P < 2.92 × 10⁻^4^; 0.05/171) (**Figure 1A**, **Supplementary Table 2**), nor did any of the 23 lipid traits (P < 2.17 × 10⁻³) (**Figure 1B**, **Supplementary Table 3**). However, three metabolites—two fatty acid-related and one amino acid/other—showed significant associations. Higher DHAbyFA [OR = 1.26, 95% CI: 1.10 – 1.43, P = 5.58 × 10⁻^4^] and FAw3byFA [OR = 1.22, 95% CI: 1.13 – 1.31, P = 2.51 × 10⁻^7^] levels were associated with increased CRC risk (Bonferroni cutoff: P < 2.78 × 10⁻³) (**Figure 1C**, **Supplementary Table 4**). Conversely, lower pyruvate (Pyr) levels acted as a protective factor against CRC [OR (95% CI) = 0.59 (0.49 – 0.70), P = 6.81 × 10⁻^9^], passing the corrected threshold for the amino acid/other group (P < 2.63 × 10⁻³) (**Figure 1D**, **Supplementary Table 5**). Robustness checks (weighted median, Egger, IVW radial, RAPS) confirmed the direction and significance of effects (**Figures 2A, B**). RAPS estimates aligned with IVW: DHAbyFA (OR = 1.28, P = 5.35 × 10⁻^5^), FAw3byFA (OR = 1.22, P = 3.32 × 10⁻^5^), and Pyr (OR = 0.58, P = 4.52 × 10⁻³).

No significant pleiotropy or heterogeneity was detected for these metabolites (P > 0.05; **Table 1**), supporting valid instruments. One outlier SNP (rs4601123) was identified in DHAbyFA-CRC analysis (**Figure 2C**). After removing it, causal associations remained consistent across methods, with no residual pleiotropy (**Figure 2D**). Colocalization analysis showed strong evidence for shared loci with CRC for FAw3byFA (PP.H4 ≈ 0.97), but not for DHAbyFA (PP.H3 ≈ 0.81) or Pyr (PP.H1 ≈ 0.72), suggesting the latter two act via independent loci (**Figures 2E, F**).

**Table 1:** Sensitive analysis for significant causal relationship between metabolites and CRC.

Metabolites	Heterogeneity			Pleiotropy		
	Method	Q	Q_P value	Egger_intercept	Standard error	P value
Ratio of 22:6 docosahexaenoic acid to total fatty acids (DHAbyFA)	MR Egger	15.205	0.364	-0.014	0.008	0.096
	IVW	18.656	0.230	-	-	-
Ratio of omega-3 fatty acids to total fatty acids (FAw3byFA)	MR Egger	5.726	0.929	-0.013	0.008	0.108
	IVW	8.740	0.792	-	-	-
Pyruvate levels (Pyr)	MR Egger	0.205	0.651	0.019	0.033	0.675
	IVW	0.519	0.772	-	-	-

Together, these findings suggest FAw3byFA as a likely causal biomarker for CRC, while DHAbyFA and Pyr may be biologically relevant but genetically distinct contributors.

### Immune cell-mediated metabolite–CRC causal pathway

**Figure 3:**
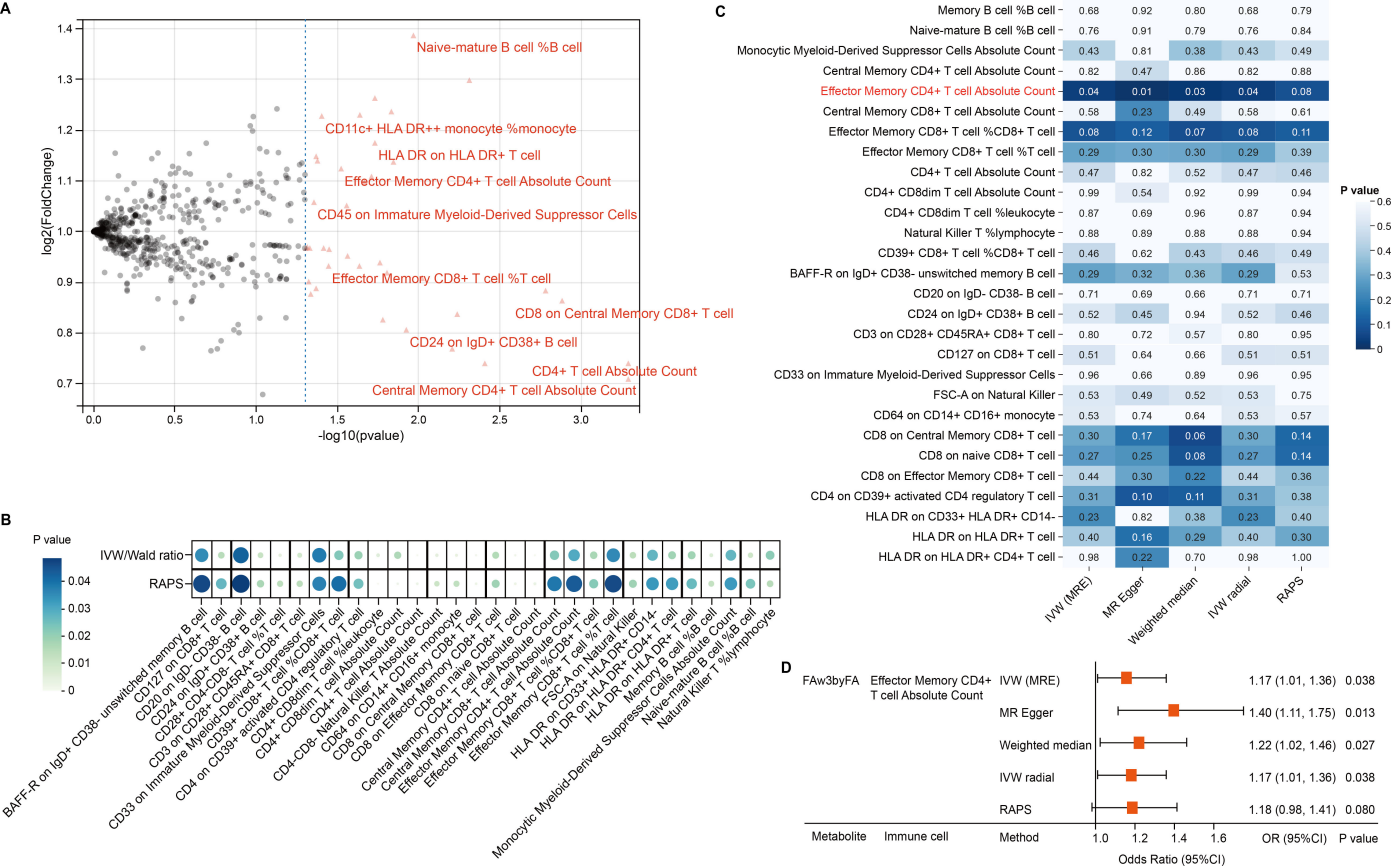
Immune mediation of omega-3 effects on CRC. **(A)** Volcano plot showing immune trait associations with CRC. **(B)** Bubble plot of 30 candidate traits across robust MR methods. **(C)** Heatmap of MR significance for FAw3byFA → immune traits. **(D)** Forest plot confirms CD4^+^ effector memory T-cell mediation.

Given the immune system’s central role in CRC pathogenesis, we next tested whether genetically predicted immune cell traits mediate the metabolite–CRC link. IVs were identified for 731 immune phenotypes, of which 614 met the inclusion criteria, with F-statistics ranging from 29.85 to 5062.70—indicating strong instrument validity. Thirty-nine immune traits were nominally associated with CRC (P < 0.05); 16 showed positive associations and 23 showed inverse associations (**Figure 3A**, **Supplementary Table 6**). RAPS-based MR confirmed 30 associations: 18 protective and 12 risk-related phenotypes (**Figure 3B**, **Table 2**, **Supplementary Table 7**).

**Table 2:** Robust causal estimates of immune cell phenotype and CRC risk.

Exposure	Outcome	method	nsnp	OR	OR_LCI	OR_UCI	P value
BAFF-R on IgD+ CD38- unswitched memory B cell	CRC	RAPS	7	0.967	0.935	1.000	0.047
		IVW	7	0.966	0.935	0.998	0.036
CD127 on CD8+ T cell	CRC	RAPS	1	1.236	1.024	1.493	0.027
		Wald ratio	1	1.236	1.043	1.466	0.015
CD20 on IgD- CD38- B cell	CRC	RAPS	4	0.886	0.786	0.999	0.049
		IVW	4	0.888	0.791	0.996	0.043
CD24 on IgD+ CD38+ B cell	CRC	RAPS	2	0.806	0.674	0.962	0.017
		IVW	2	0.806	0.682	0.954	0.012
CD28+ CD4-CD8- T cell %T cell	CRC	RAPS	1	1.397	1.071	1.822	0.014
		Wald ratio	1	1.397	1.107	1.762	0.005
CD3 on CD28+ CD45RA+ CD8+ T cell	CRC	RAPS	3	0.908	0.840	0.981	0.014
		IVW	3	0.907	0.843	0.977	0.010
CD33 on Immature Myeloid-Derived Suppressor Cells	CRC	RAPS	6	0.971	0.945	0.998	0.037
		IVW	6	0.968	0.940	0.998	0.038
CD39+ CD8+ T cell %CD8+ T cell	CRC	RAPS	7	0.936	0.879	0.997	0.041
		IVW	7	0.932	0.876	0.990	0.023
CD4 on CD39+ activated CD4 regulatory T cell	CRC	RAPS	4	1.109	1.013	1.213	0.025
		IVW	4	1.108	1.017	1.208	0.019
CD4+ CD8dim T cell %leukocyte	CRC	RAPS	2	0.834	0.757	0.919	0.000
		IVW	2	0.836	0.737	0.950	0.006
CD4+ CD8dim T cell Absolute Count	CRC	RAPS	2	0.821	0.724	0.931	0.002
		IVW	2	0.827	0.708	0.966	0.017
CD4+ T cell Absolute Count	CRC	RAPS	1	0.740	0.608	0.899	0.003
		Wald ratio	1	0.740	0.624	0.877	0.001
CD4-CD8- Natural Killer T Absolute Count	CRC	RAPS	1	1.298	1.062	1.587	0.011
		Wald ratio	1	1.298	1.082	1.556	0.005
CD64 on CD14+ CD16+ monocyte	CRC	RAPS	2	0.742	0.587	0.940	0.013
		IVW	2	0.740	0.603	0.908	0.004
CD8 on Central Memory CD8+ T cell	CRC	RAPS	3	0.863	0.784	0.949	0.002
		IVW	3	0.863	0.789	0.944	0.001
CD8 on Effector Memory CD8+ T cell	CRC	RAPS	4	0.919	0.856	0.986	0.019
		IVW	4	0.920	0.860	0.984	0.016
CD8 on naive CD8+ T cell	CRC	RAPS	2	0.884	0.815	0.958	0.003
		IVW	2	0.884	0.818	0.955	0.002
Central Memory CD4+ T cell Absolute Count	CRC	RAPS	1	0.709	0.563	0.893	0.003
		Wald ratio	1	0.709	0.584	0.861	0.001
Central Memory CD8+ T cell Absolute Count	CRC	RAPS	1	1.231	1.010	1.499	0.039
		Wald ratio	1	1.231	1.029	1.472	0.023
Effector Memory CD4+ T cell Absolute Count	CRC	RAPS	1	1.124	1.003	1.259	0.044
		Wald ratio	1	1.124	1.011	1.248	0.030
Effector Memory CD8+ T cell %CD8+ T cell	CRC	RAPS	4	0.939	0.889	0.991	0.022
		IVW	4	0.939	0.891	0.989	0.017
Effector Memory CD8+ T cell %T cell	CRC	RAPS	2	0.932	0.869	0.999	0.047
		IVW	2	0.932	0.872	0.995	0.036
FSC-A on Natural Killer	CRC	RAPS	3	1.139	1.022	1.269	0.019
		IVW	3	1.139	1.026	1.263	0.014
HLA DR on CD33+ HLA DR+ CD14-	CRC	RAPS	3	1.052	1.004	1.101	0.033
		IVW	3	1.052	1.006	1.100	0.028
HLA DR on HLA DR+ CD4+ T cell	CRC	RAPS	1	1.264	1.020	1.567	0.033
		Wald ratio	1	1.264	1.040	1.537	0.019
HLA DR on HLA DR+ T cell	CRC	RAPS	1	1.175	1.019	1.355	0.027
		Wald ratio	1	1.175	1.027	1.344	0.019
Memory B cell %B cell	CRC	RAPS	2	0.770	0.627	0.945	0.012
		IVW	2	0.770	0.638	0.928	0.006
Monocytic Myeloid-Derived Suppressor Cells Absolute Count	CRC	RAPS	5	0.953	0.911	0.996	0.033
		IVW	5	0.953	0.913	0.995	0.027
Naive-mature B cell %B cell	CRC	RAPS	1	1.388	1.044	1.845	0.024
		Wald ratio	1	1.388	1.079	1.786	0.011
Natural Killer T %lymphocyte	CRC	RAPS	7	1.111	1.021	1.209	0.015
		IVW	7	1.098	1.014	1.190	0.022

nsnp, number of single nucleotide polymorphism; OR, odds ratio; OR_LCI, Odds ratio 95% lower confidence interval; OR_UCI, Odds ratio 95% upper confidence interval.

We then conducted two-step MR to explore mediation by immune cells in the FAw3byFA–CRC relationship. In step 1, 30 immune phenotypes with confirmed CRC links were retained. In step 2, IVW MR found FAw3byFA to be positively associated with Effector Memory CD4^+^ T cell Absolute Count (β = 0.16, P = 0.03), showing nominal significance (**Figure 3C**, **Supplementary Table 8**). Sensitivity methods, including MR-Egger (β = 0.33, P = 0.01), supported this result (**Figures 3C, D**, **Supplementary Table 9**). No evidence of heterogeneity or pleiotropy was observed (**Supplementary Tables 10**, **11**), reinforcing the robustness of the findings.

These results suggest that increased FAw3byFA levels are genetically linked to elevated Effector Memory CD4^+^ T cell counts, which are independently associated with higher CRC risk. To quantify mediation, we decomposed the total effect: FAw3byFA’s total effect on CRC was β_total = 0.20, with indirect effect β_med = β_1_ × β_2_ = 0.02 (β_1_ = 0.12 for immune cell to CRC; β_2_ = 0.16 for FAw3byFA to immune cell), and a direct effect of β_direct = 0.18. This indicates a mediation proportion of 10% (0.02/0.20).

Overall, these data identify Effector Memory CD4^+^ T cell Absolute Count as a partial genetic mediator of FAw3byFA’s causal effect on CRC, accounting for a quantifiable share of its impact.

### Genetically predicted omega-3-related methylation sites increase CRC risk

**Figure 4:**
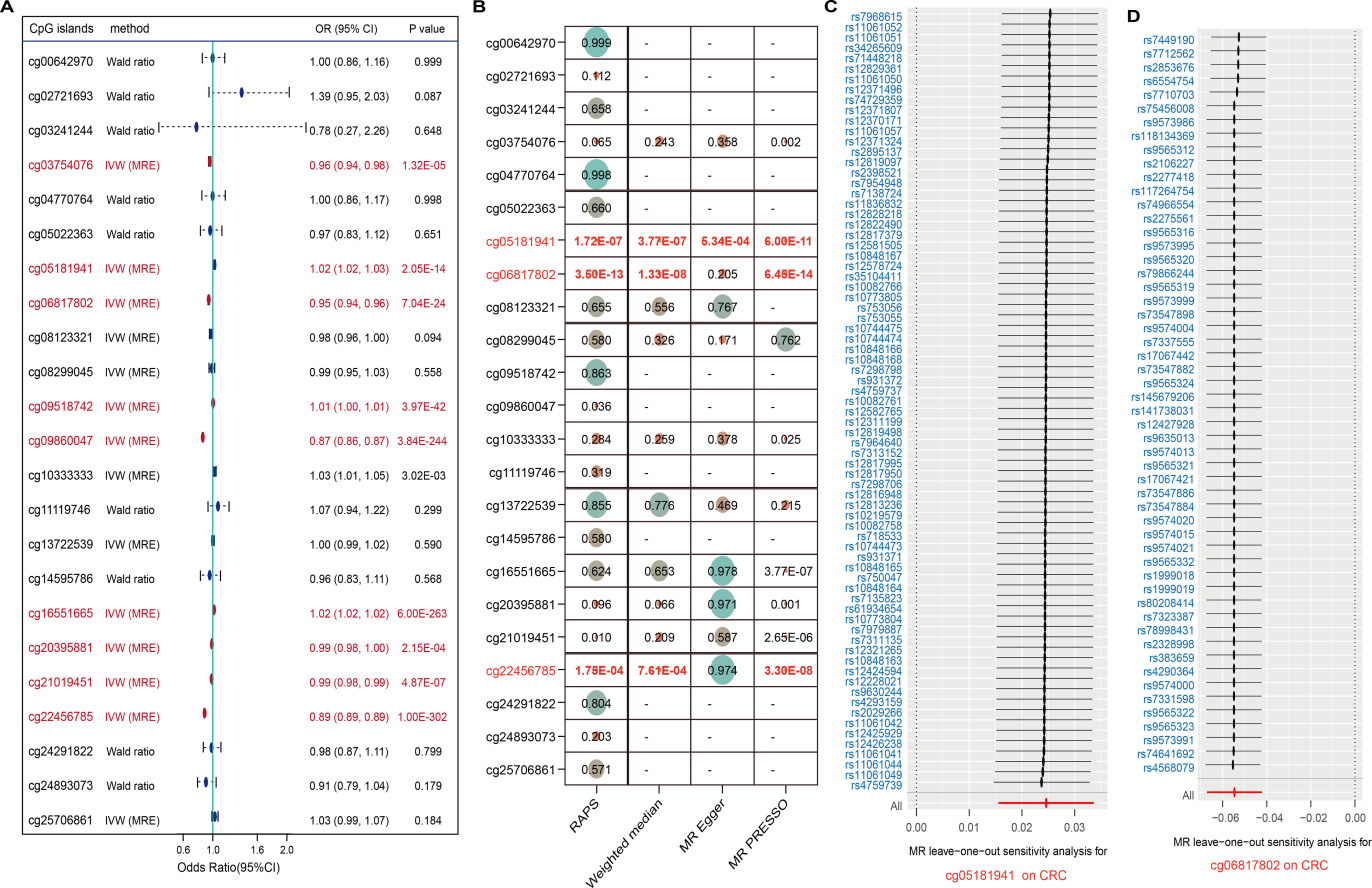
CpG methylation sites linked to omega-3 and CRC. **(A)** Forest plot of 23 CpG sites; significant ones in red. **(B)** Robustness checks with four MR methods. **(C, D)** Leave-one-out analyses confirm reliability for two CpGs.

Recent studies have highlighted that DNA methylation, as an epigenetic modification, significantly influences metabolite levels and may consequently disrupt gene expression and contribute to disease pathogenesis. In this study, we investigated the EWAS findings for omega-3 PUFAs and performed epigenetic MR analysis. A total of 47 CpG sites associated with omega-3 PUFA levels were identified (**Supplementary Table 12**). From the ARIES mQTL database, we further identified 996 SNPs representing mQTLs associated with 29 of these CpG sites, among which 23 were available for outcome analysis (**Supplementary Table 13**). Using IVW and Wald ratio methods, we found that nine CpG sites exhibited causal associations with CRC, surpassing the multiple testing correction threshold (P < 0.002; 0.05/23) (**Figure 4A**). Additional analyses using RAPS, weighted median, and MR-PRESSO methods consistently highlighted strong causal links for cg05181941, cg06817802, and cg22456785. Specifically, cg22456785 (OR = 0.89, 95% CI: [0.89 – 0.89], P = 1.00E - 302) and cg06817802 (OR = 0.95, 95% CI: [0.94 – 0.96], P = 7.04E - 24) were found to be protective, whereas cg05181941 (OR = 1.02, 95% CI: [1.02 – 1.03], P = 2.05E - 14) was identified as a risk factor (**Figure 4B**, **Supplementary Tables 14**, **15**). Sensitivity analyses revealed no significant heterogeneity in the MR estimates. In pleiotropy assessment, cg22456785 showed no evidence of horizontal pleiotropy based on the MR-Egger intercept test (P = 0.987), whereas cg05181941 and cg06817802 displayed potential pleiotropic effects (**Table 3**). To determine whether any single mQTL was disproportionately influencing the causal effect estimate, we performed a leave-one-out analysis. Results indicated that the observed associations for cg05181941 and cg06817802 were not driven by any individual SNP (**Figures 4C, D**).

**Table 3:** Sensitive analysis for significant causal relationship between omega-3 fatty acids-associated CpG islands and CRC.

CpG islands	Heterogeneity			Pleiotropy		
	Method	Q	Q_P value	Egger_intercept	Standard error	P value
cg05181941	MR Egger	28.698	0.999	-0.035	0.013	0.009
	IVW	35.979	0.999	-	-	-
cg06817802	MR Egger	14.335	0.999	-0.067	0.014	8.56E-06
	IVW	38.716	0.929	-	-	-
cg22456785	MR Egger	0.025	0.999	0.030	1.692	0.987
	IVW	0.025	0.999	-	-	-

**Figure 5:**
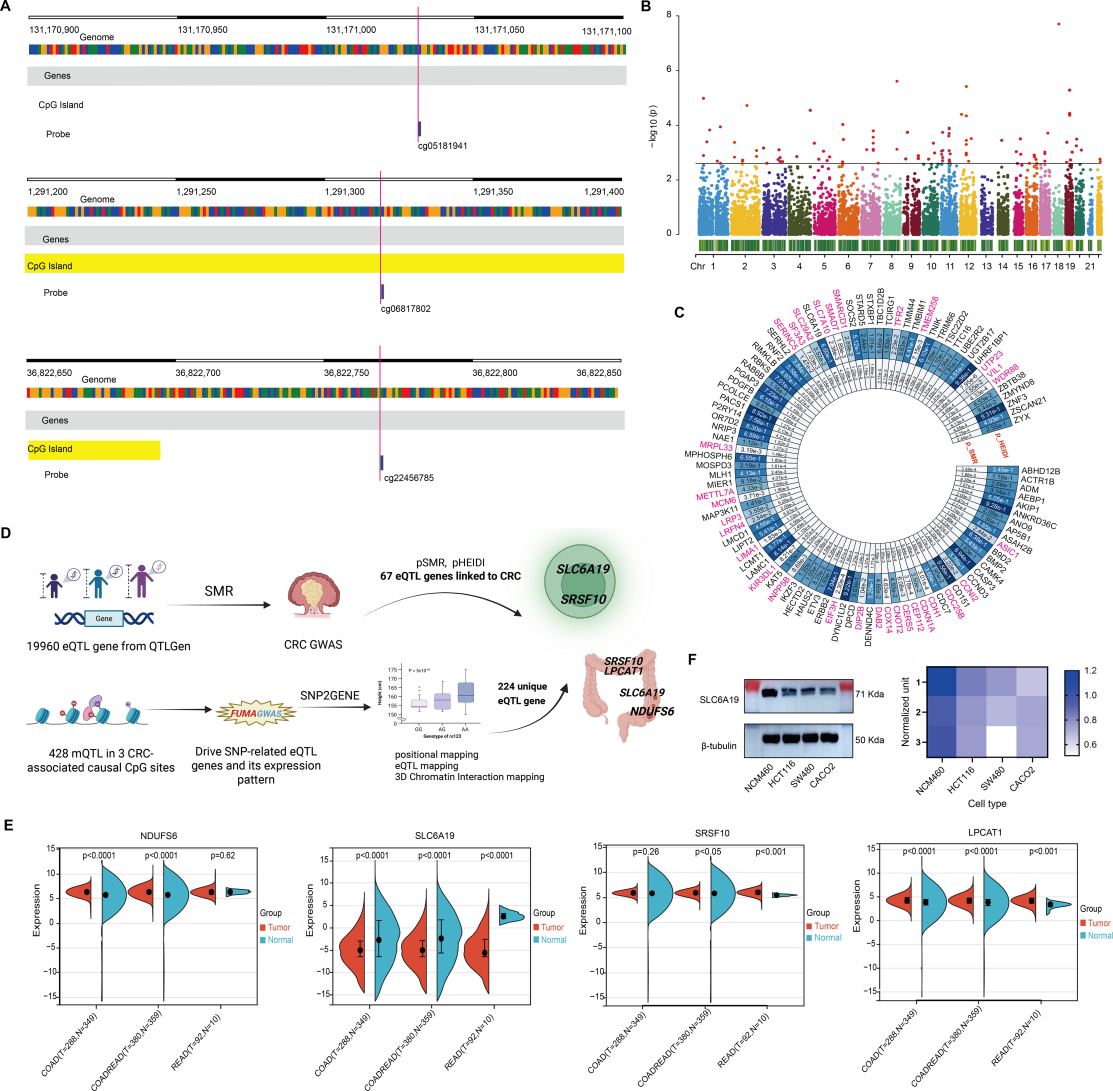
Omega 3-associated CpG sites-derived CRC candidate genes. **(A)** Genomic map of 3 significant CpGs. **(B)** Manhattan plot of SMR results for 19,960 eQTL genes. **(C)** Circular plot of 67 CRC-associated genes. **(D)** Pipeline integrating mQTL, eQTL, and CRC GWAS to identify 7 candidate genes. **(E)** TCGA-based expression comparison; SLC6A19 is consistently downregulated. **(F)** Immunoblot confirms reduced SLC6A19 protein in CRC cells.

Overall, elevated methylation at cg05181941 was associated with increased CRC risk, while lower methylation at cg06817802 and cg22456785 was linked to reduced CRC susceptibility (**Figure 5A**). These findings underscore the potential of omega-3-related DNA methylation as biomarkers for CRC susceptibility.

### Identification of CRC candidate target genes from omega-3-related CpG islands

To identify genes underlying omega-3 CpG methylation, we mapped CpG-associated mQTLs to eQTLs using transcriptomic data from 19,960 genes. SMR analysis (threshold P < 0.0025; 0.05/20) identified 98 genes (**Figure 5B**), 31 of which were excluded due to pleiotropy (HEIDI P < 0.05), leaving 67 CRC candidate genes (**Figure 5C**, **Supplementary Tables 16**, **17**).

Using FUMA’s SNP2GENE, we assessed whether mQTLs for the three significant CpGs also served as eQTLs. Among 428 mQTLs, 224 unique eQTL target genes were found across whole blood and 118 tissues (**Figure 5D**, **Supplementary Table 18**). Overlap with the 67 SMR-identified genes revealed seven shared candidates: C1QB, LPCAT1, NDUFS6, SLC6A19, SRSF10, SRSF6, and YWHAH (**Supplementary Table 19**). Four—LPCAT1, NDUFS6, SLC6A19, and SRSF10—were expressed in T cells and colon tissue, suggesting possible immune-mediated mechanisms (**Figure 5D** **;** **Table 4**).

**Table 4:** Interaction targets between priority genes identified by CPG-mQTL-associated eQTLs and SMR-defined CRC risk genes.

uniqID	db	tissue	gene	testedAllele	P value	signed_stats	chr	pos	symbol
5:1287505:C:T	eQTLcatalogue	CEDAR_T-cell_CD4	ENSG00000174358	T	0.039	0.028	5	1287505	SLC6A19
5:1289975:A:C	eQTLcatalogue	CEDAR_T-cell_CD4	ENSG00000174358	A	0.014	0.030	5	1289975	SLC6A19
5:1291331:A:AC	eQTLcatalogue	CEDAR_T-cell_CD4	ENSG00000174358	A	0.013	0.031	5	1291331	SLC6A19
5:1296072:A:G	eQTLcatalogue	CEDAR_T-cell_CD4	ENSG00000174358	A	0.014	0.031	5	1296072	SLC6A19
5:1297258:C:T	eQTLcatalogue	CEDAR_T-cell_CD4	ENSG00000174358	T	0.043	0.028	5	1297258	SLC6A19
5:1297918:C:T	eQTLcatalogue	CEDAR_T-cell_CD4	ENSG00000174358	T	0.018	0.029	5	1297918	SLC6A19
1:23703768:A:G	eQTLcatalogue	CEDAR_T-cell_CD8	ENSG00000188529	A	0.008	-0.308	1	23703768	SRSF10
1:23703768:A:G	eQTLcatalogue	CEDAR_T-cell_CD8	ENSG00000188529	A	0.003	-0.129	1	23703768	SRSF10
1:23703768:A:G	eQTLcatalogue	CEDAR_transverse_colon	ENSG00000188529	A	0.026	0.073	1	23703768	SRSF10
5:1288547:C:T	GTEx/v8	Colon_Sigmoid	ENSG00000145494	C	0.042	-0.064	5	1288547	NDUFS6
5:1297918:C:T	GTEx/v8	Colon_Sigmoid	ENSG00000174358	C	0.032	0.152	5	1297918	SLC6A19
5:1296072:A:G	GTEx/v8	Colon_Transverse	ENSG00000153395	G	0.007	-0.090	5	1296072	LPCAT1
5:1297258:C:T	GTEx/v8	Colon_Transverse	ENSG00000153395	C	0.007	-0.094	5	1297258	LPCAT1
5:1297918:C:T	GTEx/v8	Colon_Transverse	ENSG00000153395	C	0.005	-0.095	5	1297918	LPCAT1
5:1287505:C:T	GTEx/v7	Colon_Sigmoid	ENSG00000145494	C	0.048	-0.141	5	1287505	NDUFS6
5:1296072:A:G	GTEx/v7	Colon_Sigmoid	ENSG00000174358	G	0.036	0.247	5	1296072	SLC6A19
5:1297258:C:T	GTEx/v7	Colon_Sigmoid	ENSG00000174358	C	0.022	0.297	5	1297258	SLC6A19
5:1297918:C:T	GTEx/v7	Colon_Sigmoid	ENSG00000174358	C	0.017	0.283	5	1297918	SLC6A19
5:1296072:A:G	GTEx/v6	Colon_Transverse	ENSG00000153395	G	0.029	-2.215	5	1296072	LPCAT1
5:1297258:C:T	GTEx/v6	Colon_Transverse	ENSG00000153395	C	0.015	-2.474	5	1297258	LPCAT1

uniqID: the unique identifier for each SNP; db: source database of the eQTL; tissue: tissue or cell type where the eQTL was found; gene: Ensembl Gene ID linked to the eQTL; testedAllele: allele tested for expression association; P value: significance of the eQTL association; signed_stat: effect size or direction of association; ch: chromosome number; pos: SNP genomic position.

### Tumor phenotypes induced by CRC candidate genes

Differential expression analysis showed consistent upregulation of LPCAT1, NDUFS6, and SRSF10, while SLC6A19 was markedly downregulated in COAD, READ, and COADREAD subtypes. Notably, SLC6A19, which encodes an amino acid transporter, showed the most significant reduction across CRC types and was expressed in both colon and T cells (**Figures 5D, E**). Immunoblotting in CRC cell lines (HCT116, SW480, CACO2) confirmed reduced SLC6A19 protein levels compared to normal epithelial cells (NCM460) (**Figure 5F**), suggesting a potential functional role in CRC development.

### Low expression of SLC6A19 promotes CRC malignant phenotypes

**Figure 6:**
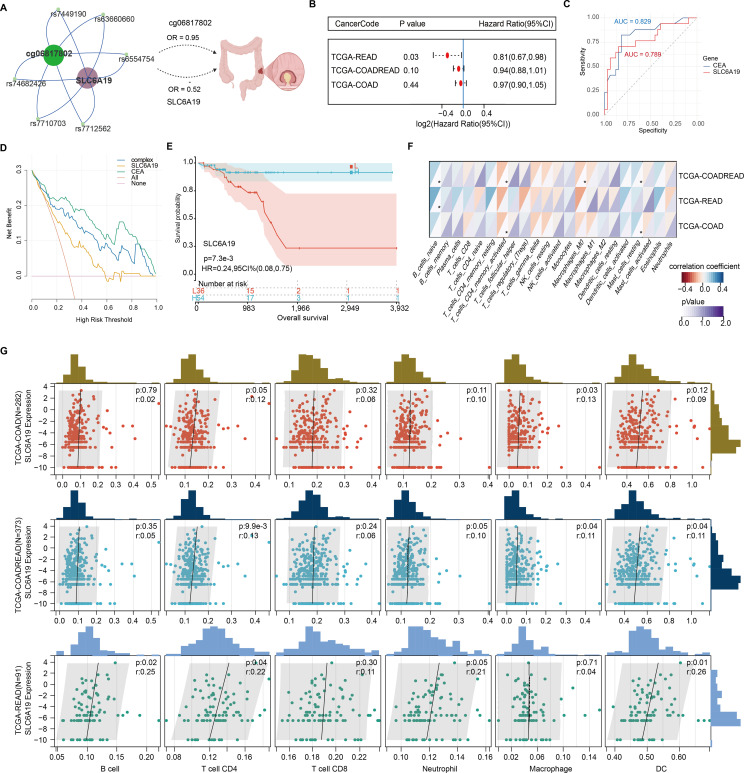
Prognostic and immunological relevance of SLC6A19 in CRC. **(A)** Illustration of the genetic and epigenetic relationship between omega-3-associated CpG site cg06817802, shared SNPs serve as both mQTL and eQTL, and SLC6A19, highlighting a potential causal axis from methylation to CRC risk. **(B)** Cox proportional hazards analysis showing that low SLC6A19 expression is significantly associated with poor prognosis in the TCGA-READ cohort. **(C)** Receiver operating characteristic (ROC) curves comparing the diagnostic performance of SLC6A19 and CEACAM5 (CEA) expression in distinguishing CRC from normal tissues. **(D)** Decision curve analysis (DCA) comparing the net clinical benefit of SLC6A19, CEACAM5 (CEA), a combined model (“complex”), and default strategies (“All” and “None”) across threshold probabilities in the COADREAD cohort. **(E)** Kaplan–Meier survival curve for SLC6A19 expression in the combined COADREAD cohort, confirming that lower expression correlates with worse overall survival. **(F)** Correlation heatmap with 22 immune cells. **(G)** Scatter plots for six immune cell subtypes. * P<0.05.

**Figure 7:**
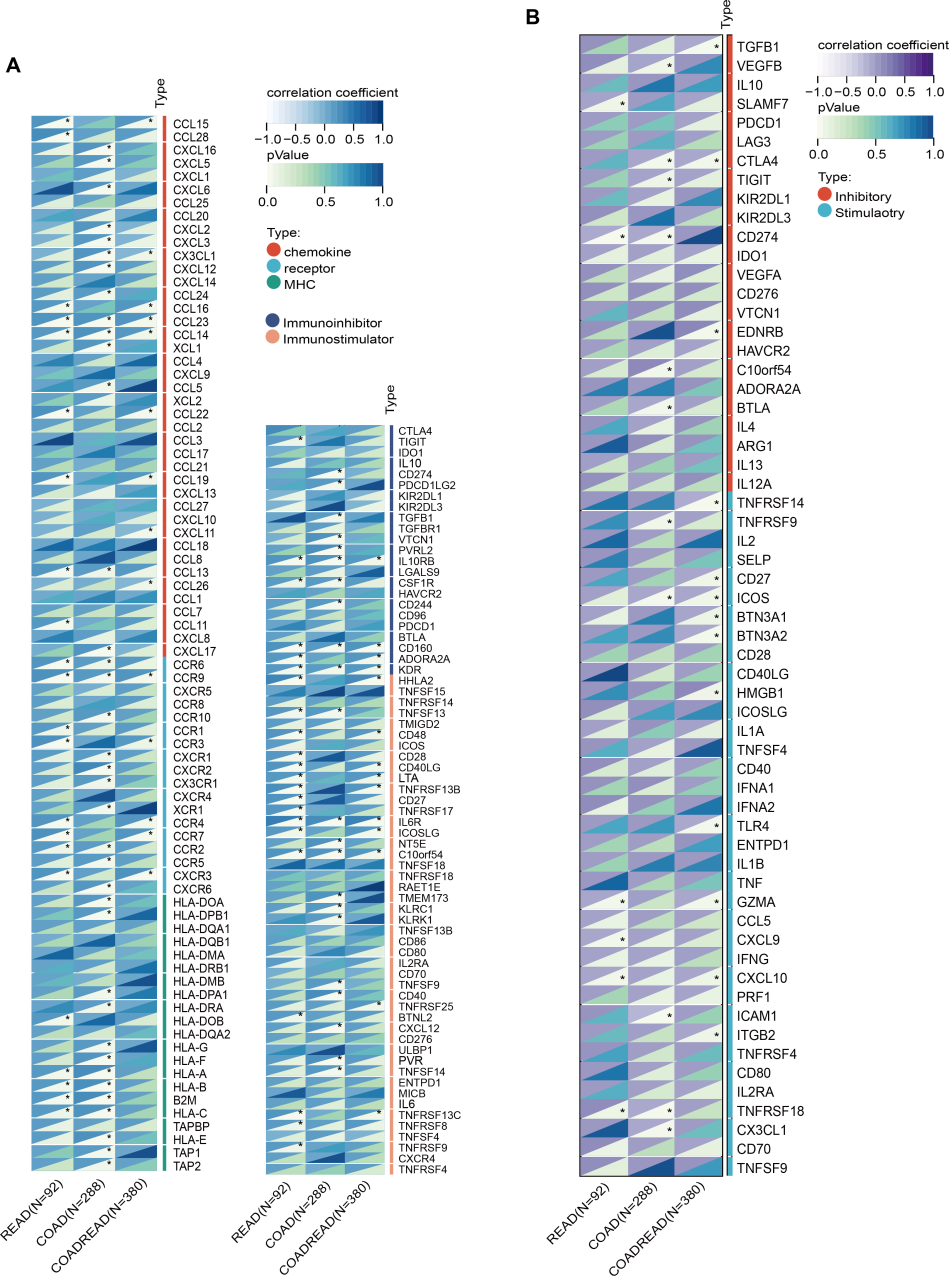
Correlation of SLC6A19 expression with immune regulatory signatures in CRC. **(A)** Heatmap showing the correlation between SLC6A19 expression and immune-related genes involved in chemokine signaling, MHC molecules, immune receptors, immunoinhibitors, and immunostimulators across TCGA-READ, COAD, and COADREAD cohorts. **(B)** Heatmap of correlations between SLC6A19 expression and 60 immune checkpoint genes, categorized as stimulatory or inhibitory. * P<0.05.

SLC6A19 was identified as a causal CRC gene via SMR and mapped to the omega-3-linked CpG site cg06817802, sharing common mQTL/eQTL SNPs (**Figure 6A**, **Supplementary Table 20**). Cox survival models showed that low SLC6A19 expression correlated with poorer outcomes in READ (HR = 0.81, P = 0.03) (**Figure 6B**). ROC analysis showed that both SLC6A19 and the established CRC biomarker CEA had moderate diagnostic accuracy for distinguishing CRC from normal tissues, with CEA performing slightly better (AUC = 0.829 vs. 0.789) in the READ cohort (**Figure 6C**). DCA indicated that the combined SLC6A19-CEA model provided greater net clinical benefit than either marker alone across most threshold probabilities (**Figure 6D**). Consistently, log-rank survival analysis confirmed that patients with low SLC6A19 expression had markedly worse survival outcomes (HR = 0.24, 95% CI: 0.08 – 0.752, P = 7.3 × 10⁻³) (**Figure 6E**). Building on prior mediation findings implicating CD4^+^ T cells in the omega-3–CRC pathway, we analyzed immune cell infiltration. Using TIMER and CIBERSORT, SLC6A19 expression was positively associated with activated CD4^+^ memory T cells in COAD and COADREAD (**Figure 6F**) and correlated with CD4^+^ T cell infiltration in all subtypes (r = 0.12 – 0.22) (**Figure 6G**). In addition, we investigated the association between SLC6A19 expression and immune regulation. Pearson correlation analyses were performed between SLC6A19 and 150 immune pathway marker genes (representing chemokine, receptor, MHC, immunoinhibitor, and immunostimulator classes), as well as 60 immune checkpoint genes (24 inhibitory and 36 stimulatory). The results indicated varying degrees of expression correlation across CRC subtypes, suggesting that SLC6A19 is broadly involved in immune modulation (**Figure 7**). Together, these data support a link between SLC6A19 expression and CD4^+^ T cell activity, reinforcing its role in the omega-3–CRC axis.

### Functional validation of SLC6A19 in CRC progression

**Figure 8:**
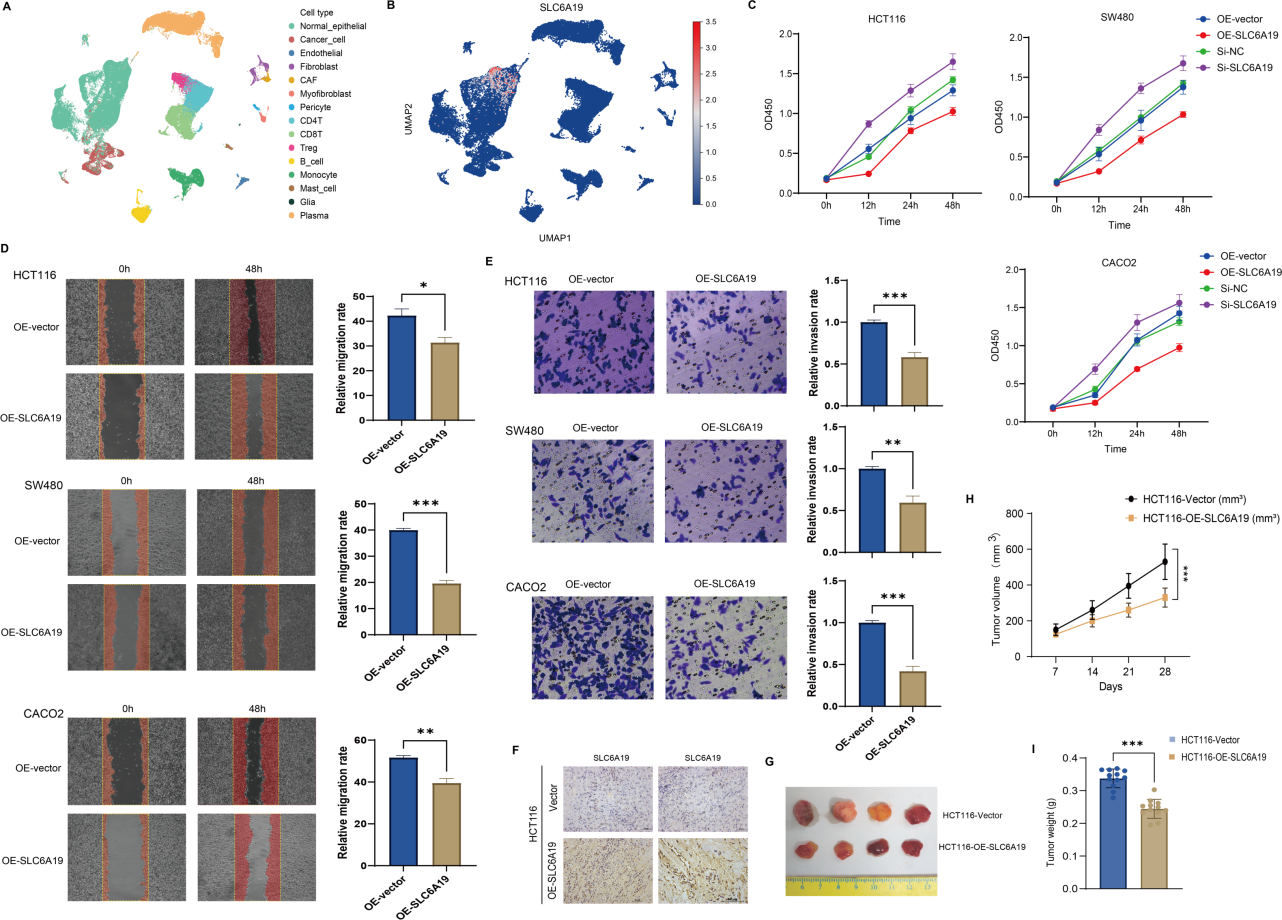
SLC6A19 restrains CRC cell growth, motility, and tumor formation. **(A)** UMAP projection of single-cell RNA-seq dataset GSE166555 illustrating the major cell populations present in human CRC tissue. **(B)** Feature plot showing the distribution and intensity of SLC6A19 expression within the same UMAP space. **(C)** CCK-8 assays: overexpression reduces proliferation; knockdown increases it. **(D)** Wound healing: migration suppressed by SLC6A19 overexpression. **(E)** Transwell invasion assays in HCT116, SW480, and CACO2 cells showing reduced invasive ability following SLC6A19 overexpression. **(F)** Immunohistochemical staining of xenograft sections verifies higher SLC6A19 protein levels in tumors derived from OE-SLC6A19 cells relative to vector controls. **(G)** Representative photographs of excised xenograft tumors (day 28). **(H)** Growth curves of tumor volume measured weekly show a marked attenuation of *in vivo* growth in the OE-SLC6A19 group. **(I)** Bar chart of final tumor weights at sacrifice corroborates the volume data. Representative images and bar graphs show significantly lower invasion rates relative to vector controls (* P < 0.05, ** P < 0.01, *** P < 0.001).

To explore what SLC6A19 might be doing in colorectal cancer, we first looked at its expression at single-cell resolution. We found that the gene shows up mainly in normal epithelial cells, and also in specific subsets of T cells within the tumor microenvironment (**Figures 8A, B**). To validate its function, we overexpressed and silenced SLC6A19 in HCT116, SW480, and CACO2 cells. CCK - 8 assays showed that overexpression inhibited proliferation, while knockdown enhanced it (**Figure 8C**). Wound healing and Transwell assays revealed reduced migration and invasion upon overexpression. Specifically, wound closure was significantly delayed in all lines (**Figure 8D**). Similarly, in the Transwell invasion assays, SLC6A19 overexpression significantly impaired the invasive capacity of CRC cells. The number of cells traversing the Matrigel-coated membrane was notably reduced. Quantitative data revealed significantly lower invasion rates in HCT116 (P < 0.001), SW480 (P < 0.01), and CACO2 (P < 0.001) cells (**Figure 8E**). We then tested this in mice. HCT116 cells with stable SLC6A19 overexpression, or empty vector controls, were implanted into BALB/c nude mice. Tumors in the SLC6A19 group showed stronger SLC6A19 staining in immunohistochemistry (**Figure 8F**) and reduced tumor size (**Figure 8G**). Tumors in the overexpression group grew more slowly (**Figure 8H**), were lighter at the endpoint (**Figure 8I**). These findings confirm a tumor-suppressive role for SLC6A19 in CRC.

## Discussion

Our integrative multi-omics analysis identifies a critical metabolic–epigenetic–transcriptional–immunological axis influencing CRC susceptibility. By combining GWAS, EWAS, transcriptomics, metabolomics, immunophenotyping, and QTL mapping, along with MR, colocalization, transcriptome-wide association, and functional validation, we reveal SLC6A19 as a functionally relevant tumor suppressor. Its reduced expression in CRC tissues and CD4^+^ T cells implicate omega-3 PUFA-mediated epigenetic repression in compromised tumor immunosurveillance.

Traditionally, omega-3 PUFAs have been considered protective due to their anti-inflammatory properties (14, 25, 40), our findings suggest a more nuanced role in CRC. Increased genetically predicted omega-3 levels (FAw3byFA, especially DHA) correlated with elevated CRC risk, challenging traditional assumptions. These results align with studies showing the pro-carcinogenic impact of an imbalanced n-6/n-3 PUFA ratio (20, 41–45). Unlike observational studies susceptible to confounding, MR reflects lifelong exposure shaped by genetics (46–49). Additionally, this contradiction may arise from the complex roles of immune regulation. For instance, although omega-3 PUFAs can suppress pro-inflammatory cytokines, they may also modulate T-cell differentiation and function, potentially affecting tumor immunity (15, 16, 18, 50). This underscores the notion that the biological effect of omega-3 fatty acids is not always protective and can differ according to tissue type, dose, inflammation status, and genetic component. Furthermore, omega-3 PUFA-derived metabolites, such as resolvins and protectins, are essential for the resolution of inflammation (17). Abnormalities in these metabolites can impair immune homeostasis and create an immunosuppressive microenvironment for tumor growth (17). Notably, FAw3byFA increased effector memory CD4^+^ T cells, implicated in tumor progression (51), with mediation analysis attributing ~10% of CRC risk to this pathway—potentially via T-cell exhaustion (51).

SLC6A19, a sodium-dependent neutral amino acid transporter in intestinal epithelium, emerged from SMR analysis of omega-3-linked CpG (cg06817802) and eQTL data. It was downregulated in CRC tissues/cell lines and inversely correlated with tumor aggressiveness. Functional assays confirmed tumor-suppressive effects, with overexpression reducing proliferation, migration, and invasion. SLC6A19 expression positively correlated with CD4^+^ T-cell infiltration, especially memory-activated subsets, aligning with its mediatory role. Mechanistically, SLC6A19 downregulation may impair glutamine/leucine transport—key to T-cell metabolism and epithelial cell homeostasis (52, 53). The cg06817802 site showed hypomethylation in protective alleles, suggesting omega-3-driven repression. Shared mQTL/eQTL variants further support a genetically anchored regulatory axis. Its link to immune checkpoint expression also hints at broader immunoregulatory roles. From a clinic standpoint, SLC6A19 can be a prognostic CRC biomarker according to its correlation with patient survival and immune infiltration patterns. Its immunomodulatory and tumor suppressor functions also render it a prospective target for immunometabolic therapy to recover epithelial integrity and enhance antitumor immune surveillance. From a translation perspective, SLC6A19 correlation with favorable prognosis, immune infiltration, and tumor suppression suggests possible functions in stratifying patients for immunometabolic therapy. In addition, pharmacological modulation or gene therapy approaches to recover SLC6A19 expression can theoretically enhance anti-tumor immune surveillance, particularly in those patients who have low SLC6A19 expression and low T-cell infiltration. The establishment of future drug development products and clinical investigations should explore targeting SLC6A19 as part of precision CRC treatment approaches.

Despite rigorous methodology, limitations remain. Bonferroni correction may have excluded true associations due to its conservatism. Immune phenotypes were analyzed regardless of instrument strength, and while valid under ratio/2SLS methods (54), residual pleiotropy and stratification remain possible. Another important limitation of our work is that the CRC, immune trait, and metabolomic data that we investigated originated mostly from European-ancestry individuals. Such genetic homogeneity could narrow the generalizability of our results to non-European populations, where genetics, diet, and environmental exposures vary. Such variations might exert opposing effects on both immune-related pathway and metabolite levels and, consequently, on the identified CRC associations. Extending the work through future studies involving multi-ethnic cohorts under different lifestyles and dietary exposures will be critical to confirming and expanding current results, making them more globally applicable. Because DNA methylation is dynamic, our mQTL-instrumented findings also require experimental validation. Future work will include targeted methylation editing at the implicated CpGs, bisulfite assays following omega-3 supplementation in colon epithelial and T-cell models, and tumor ***vs.*** adjacent-normal methylation profiling to confirm directionality and tissue specificity. Importantly, functional validation of the SLC6A19–CD4^+^ T-cell interaction in CRC mouse models or patient-derived organoids would further substantiate the proposed mechanistic link.

In summary, we propose that SLC6A19 connects omega-3 PUFA signaling with immune surveillance in CRC. Its loss may impair immune function and promote tumorigenesis, offering mechanistic insights into PUFA-related cancer risk and potential immunometabolic targets.

## Data Availability

The original contributions presented in the study are included in the article/**Supplementary Material**. Further inquiries can be directed to the corresponding author.
