# Supplementary figures and images for: Integrative multi-omics identification and functional validation of potential targets linking metabolism–immune–colorectal cancer causal pathway
Source: Front Immunol. 2025 Sep 8;16:1649788. doi: 10.3389/fimmu.2025.1649788 (PMC12450685; doi:10.3389/fimmu.2025.1649788)

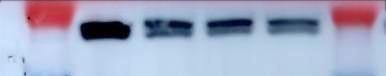

Supplement: Supplementary file 1 [file DataSheet1.zip › Repeat 1 slc crop.jpg]

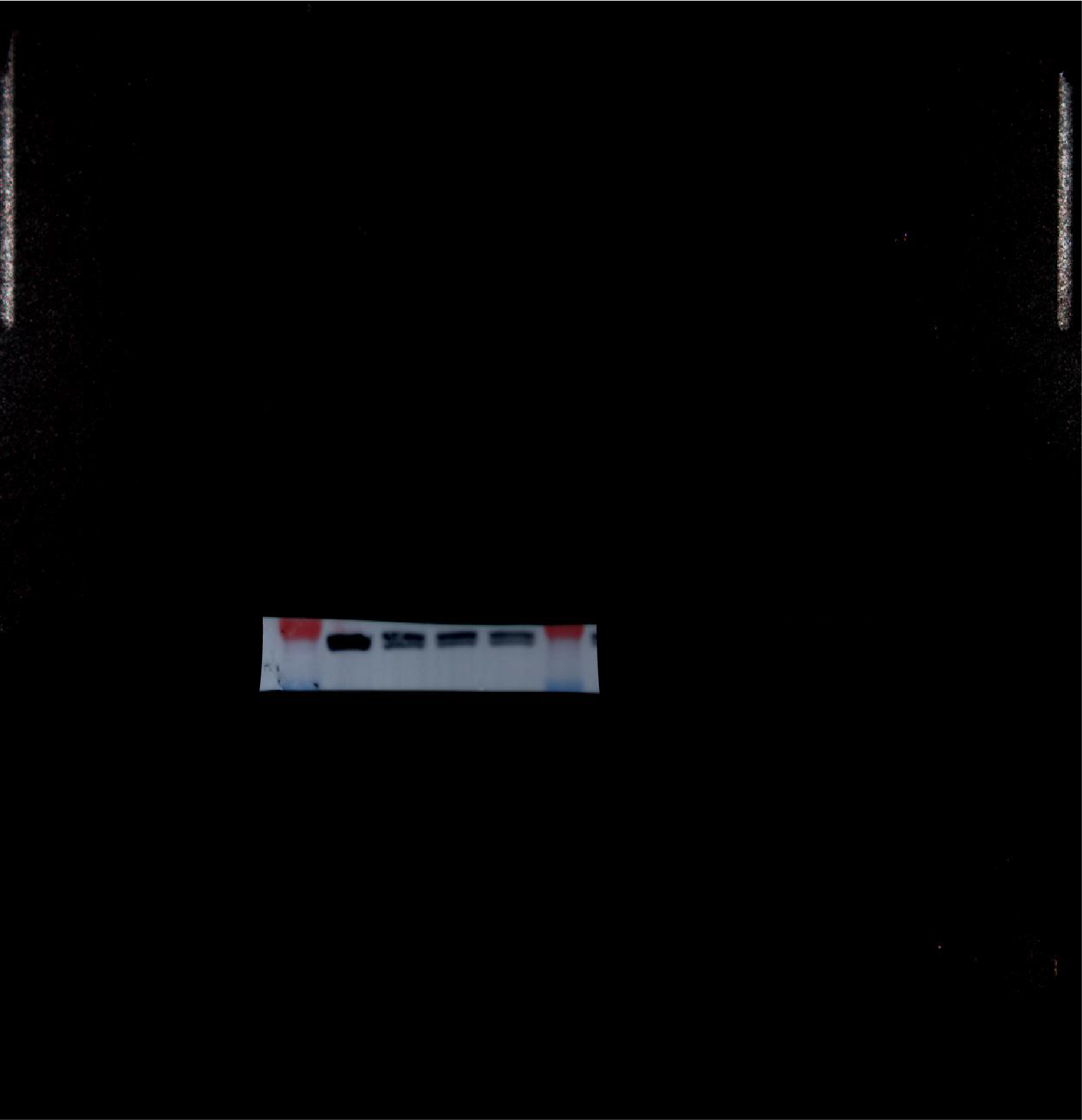

Supplement: Supplementary file 1 [file DataSheet1.zip › Repeat 1 slc.tif]

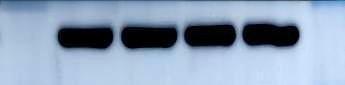

Supplement: Supplementary file 1 [file DataSheet1.zip › Repeat 1 tubu crop.jpg]

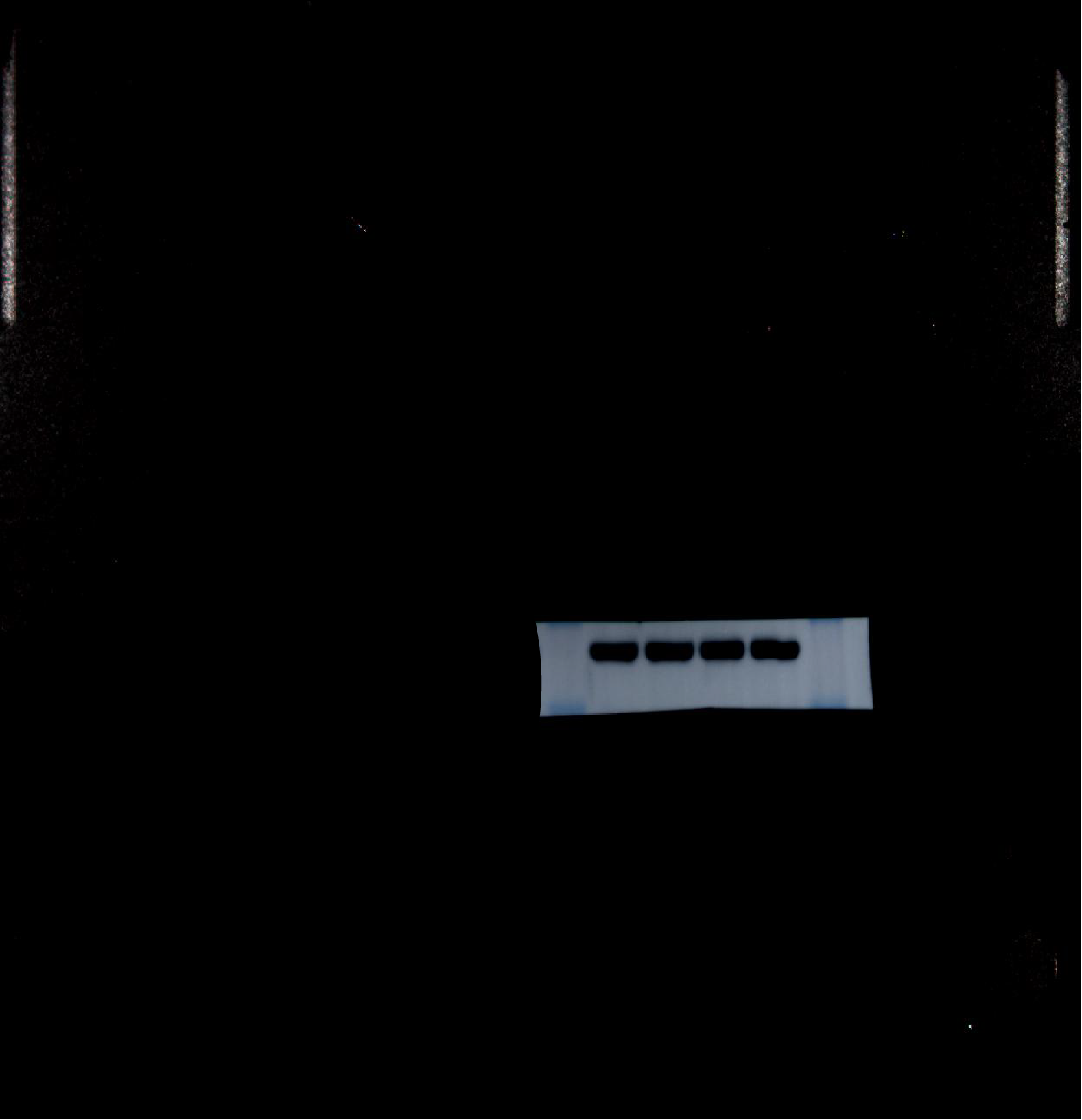

Supplement: Supplementary file 1 [file DataSheet1.zip › Repeat 1 tubu.tif]

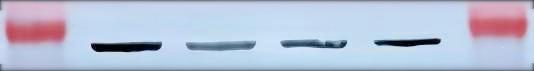

Supplement: Supplementary file 1 [file DataSheet1.zip › Repeat 2 slc crop.jpg]

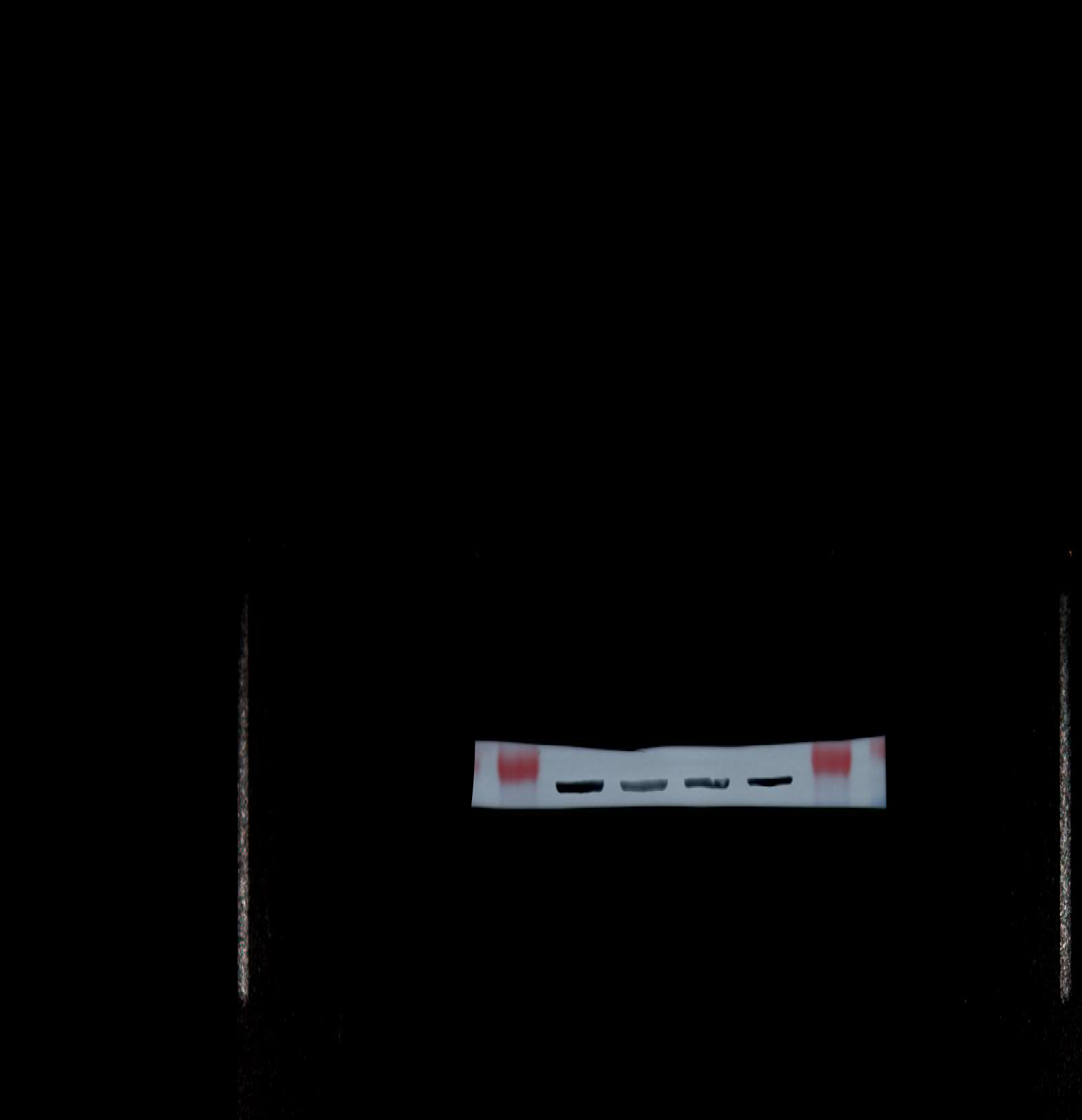

Supplement: Supplementary file 1 [file DataSheet1.zip › Repeat 2 slc.tif]

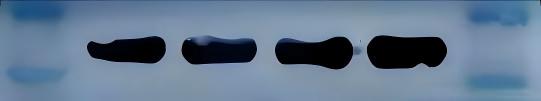

Supplement: Supplementary file 1 [file DataSheet1.zip › Repeat 2 tubu crop.jpg]

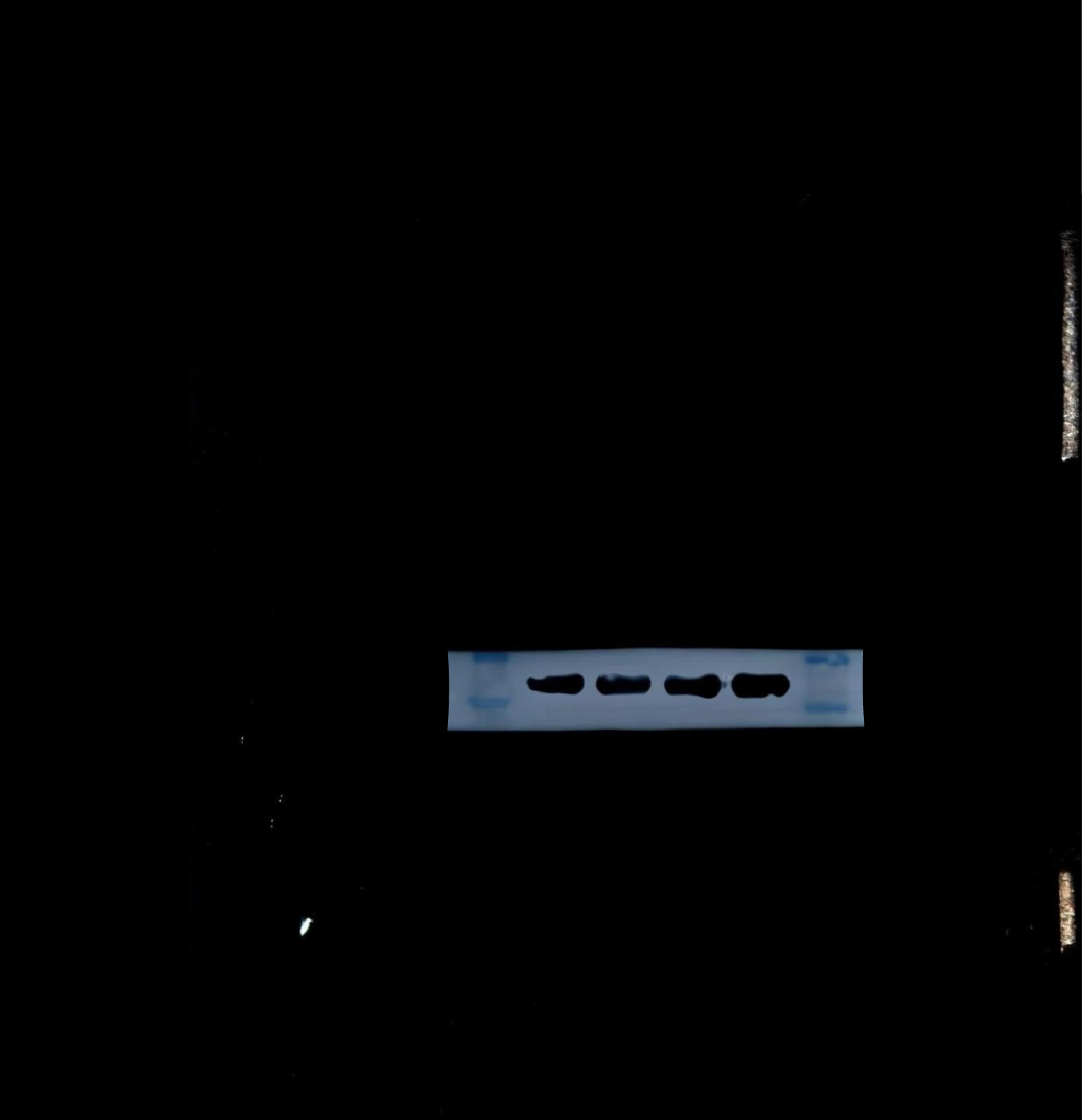

Supplement: Supplementary file 1 [file DataSheet1.zip › Repeat 2 tubu.tif]

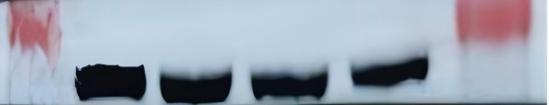

Supplement: Supplementary file 1 [file DataSheet1.zip › Repeat 3 slc crop.jpg]

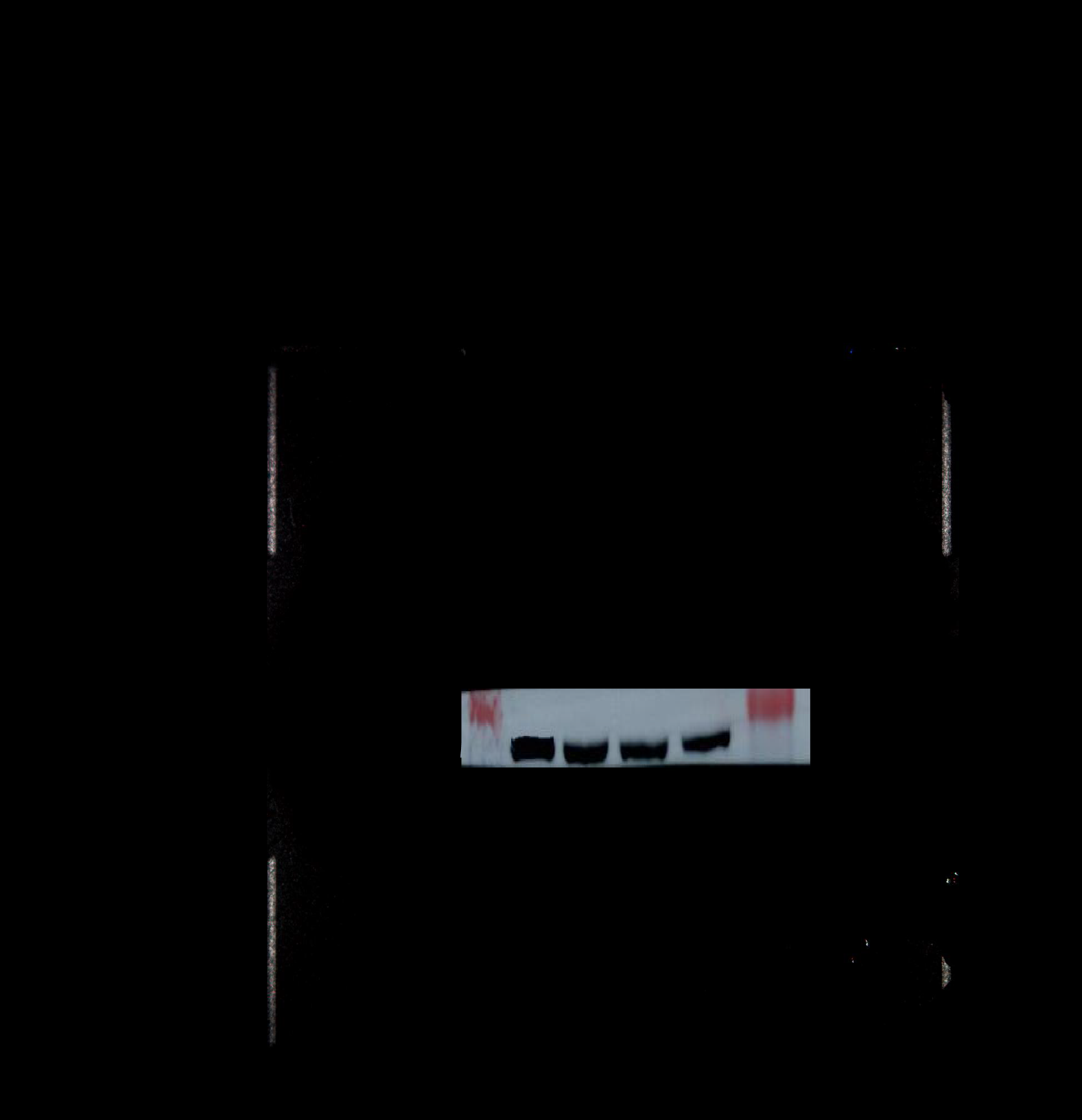

Supplement: Supplementary file 1 [file DataSheet1.zip › Repeat 3 slc.tif]

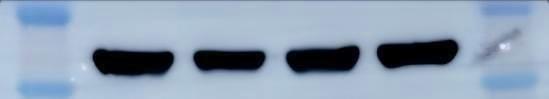

Supplement: Supplementary file 1 [file DataSheet1.zip › Repeat 3 tubu crop.jpg]

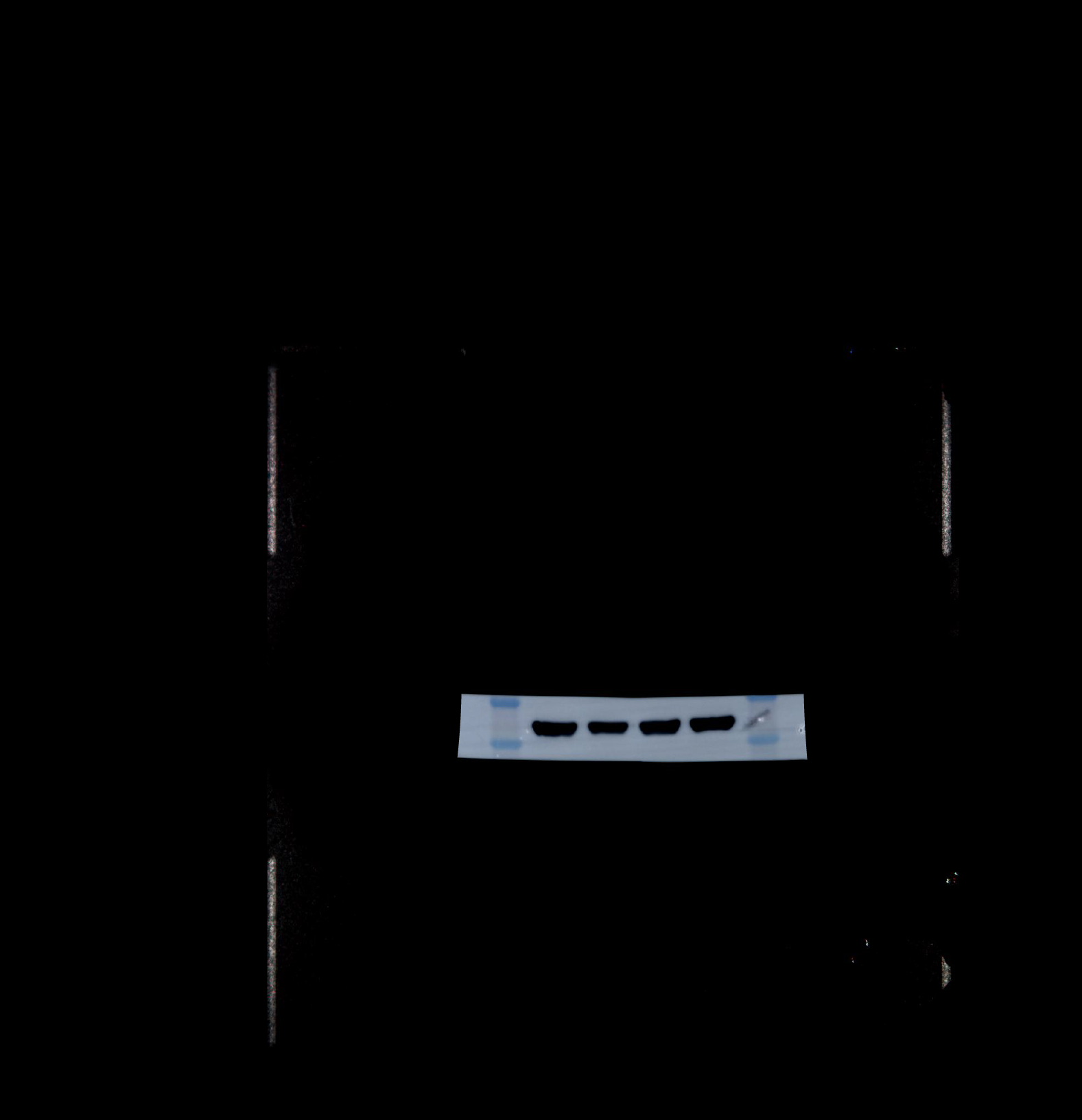

Supplement: Supplementary file 1 [file DataSheet1.zip › Repeat 3 tubu.tif]

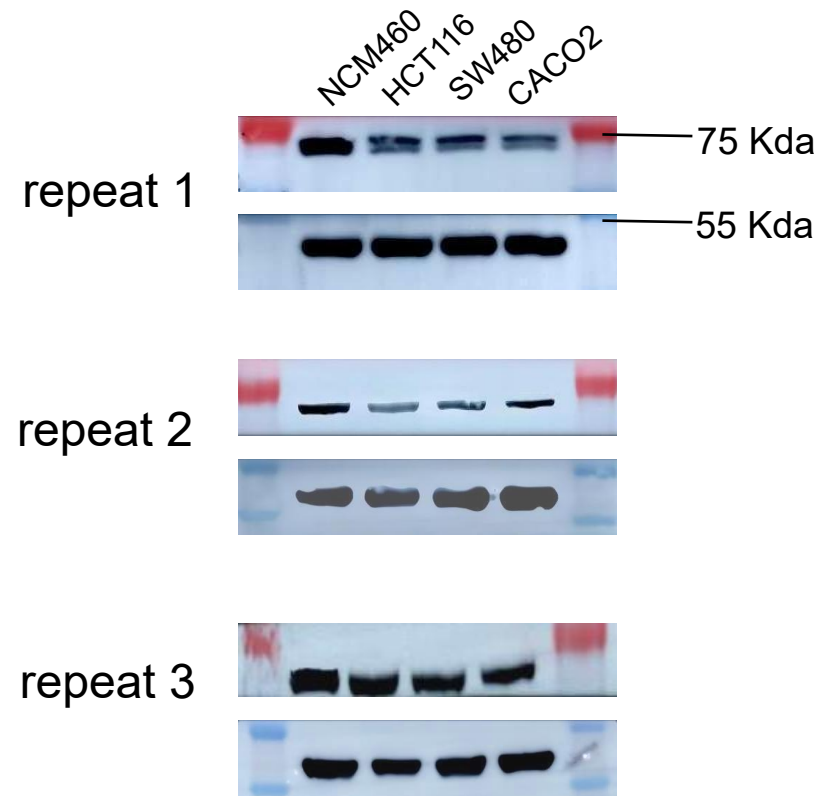

Supplement: Supplementary file 1 [file DataSheet1.zip › WB original images.pdf]

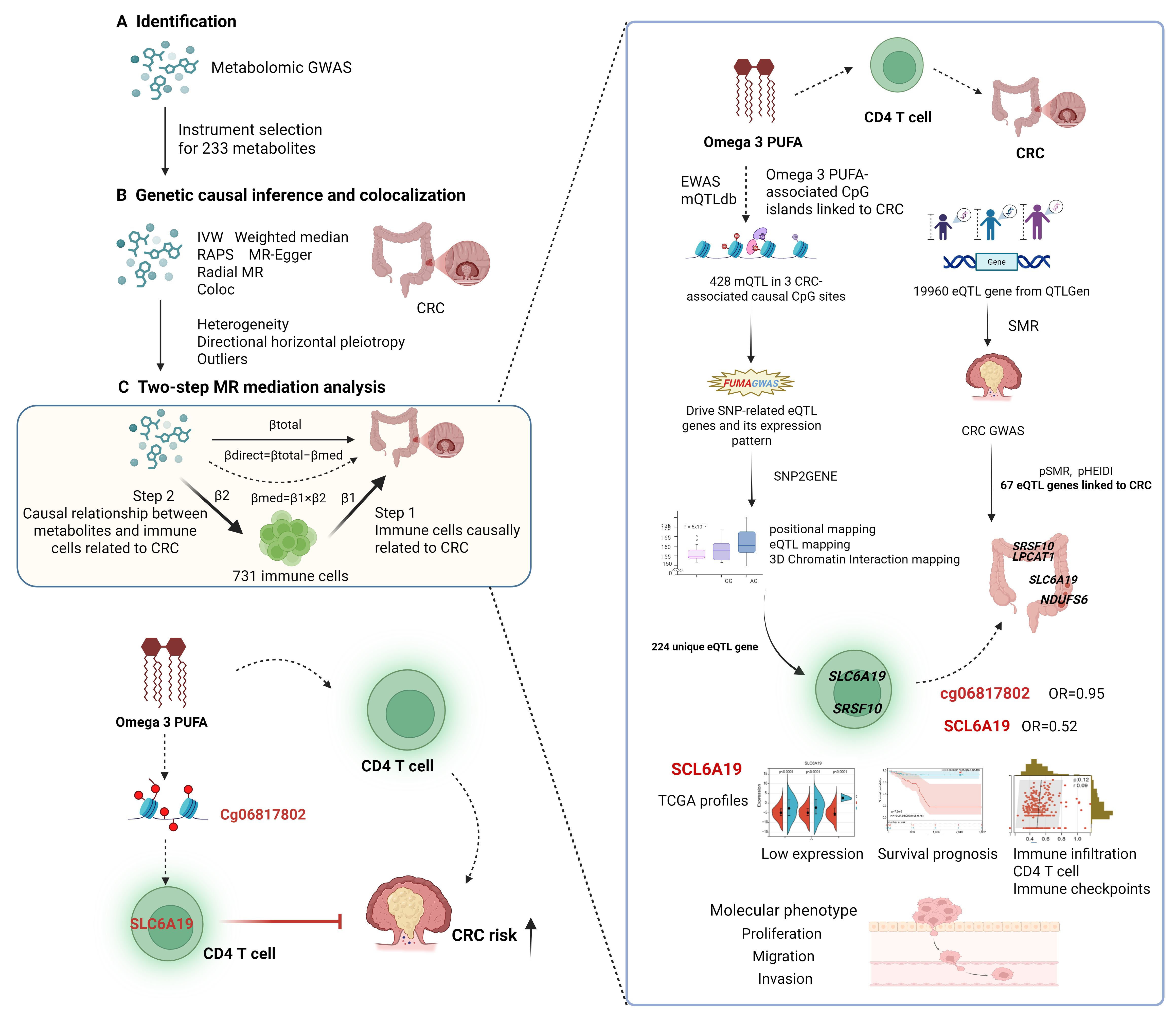

Supplement: Supplementary file 2 [file Image1.tif]
